# Transcription factor activating enhancer-binding protein 2ε (AP2ε) modulates phenotypic plasticity and progression of malignant melanoma

**DOI:** 10.1038/s41419-024-06733-3

**Published:** 2024-05-21

**Authors:** Sebastian Staebler, Ulrike Rottensteiner-Brandl, Zubeir El Ahmad, Melanie Kappelmann-Fenzl, Andreas Arkudas, Annika Kengelbach-Weigand, Anja-Katrin Bosserhoff, Sonja K. Schmidt

**Affiliations:** 1 Institute of Biochemistry, Friedrich-Alexander University Erlangen-Nürnberg (FAU), Fahrstraße 17, 91054 Erlangen, Germany; 2 Faculty of Computer Science, Deggendorf Institute of Technology, Dieter-Görlitz-Platz 1, 94469 Deggendorf, Germany; 3 Laboratory for Tissue-Engineering and Regenerative Medicine, Department of Plastic and Hand Surgery, University Hospital Erlangen-Friedrich Alexander University of Erlangen-Nürnberg FAU, 91054 Erlangen, Germany; 4 CCC Erlangen-EMN: Comprehensive Cancer Center Erlangen-EMN (CCC ER-EMN), 91054 Erlangen, Germany; 5 CCC WERA: Comprehensive Cancer Center Alliance WERA (CCC WERA), 91054 Erlangen, Germany; 6 BZKF: Bavarian Cancer Research Center (BZKF), 91054 Erlangen, Germany

**Keywords:** Melanoma, Cancer stem cells, Metastasis, Tumour heterogeneity

## Abstract

Malignant melanoma, the most aggressive form of skin cancer, is often incurable once metastatic dissemination of cancer cells to distant organs has occurred. We investigated the role of Transcription Factor Activating Enhancer-Binding Protein 2ε (AP2ε) in the progression of metastatic melanoma. Here, we observed that AP2ε is a potent activator of metastasis and newly revealed AP2ε to be an important player in melanoma plasticity. High levels of AP2ε lead to worsened prognosis of melanoma patients. Using a transgenic melanoma mouse model with a specific loss of AP2ε expression, we confirmed the impact of AP2ε to modulate the dynamic switch from a migratory to a proliferative phenotype. AP2ε deficient melanoma cells show a severely reduced migratory potential in vitro and reduced metastatic behavior in vivo. Consistently, we revealed increased activity of AP2ε in quiescent and migratory cells compared to heterogeneously proliferating cells in bioprinted 3D models. In conclusion, these findings disclose a yet-unknown role of AP2ε in maintaining plasticity and migration in malignant melanoma cells.

## Introduction

The development and progression of malignant melanoma is characterized by the de-differentiation and molecular changes in melanocytes induced by various gene defects, as well as by the de-regulation of signaling pathways and tumor-relevant transcription factors [1, 2]. Consequently, the cells develop a malignant phenotype, including increased proliferative, migratory and invasive properties, replicative immortality and enhanced induction of angiogenesis and apoptosis resistance [3]. A contribution of AP2 family members to melanoma development and progression was analyzed by several groups, including our own [4–7]. The AP2 transcription factor family consists of 5 isoforms TFAP2a (AP2α), TFAP2b (AP2β), TFAP2c (AP2γ), TFAP2d (AP2δ) and TFAP2e (AP2ε). The AP2 family members regulate gene expression via the palindromic binding sequence 5’-GCCN3GGC-3’ in the promoter of target genes and are involved in a variety of processes during embryogenesis and differentiation [8, 9]. It has already been proposed, that the specific ratio of certain AP2 isoforms is important to facilitate different cellular processes [10]. In melanoma, the role of each AP2 transcription factor is still controversial. As an example, for AP2α, either an induction of expression in melanoma or a strong reduction was observed, both of which were attributed to melanoma progression [11–13]. This isoform has also been implicated in the control of cell proliferation and survival in melanoma and in the regulation of EMT-related genes, such as E-cadherin and N-cadherin, in melanoma cells [14]. Dysregulation of AP2α may disrupt melanocyte differentiation and contribute to melanoma initiation and progression [15]. The expression of AP2γ and AP2δ has also been linked to promote proliferation of breast and prostate cancer cells, respectively [16, 17].

The use of valid, isoform-specific mouse models such as full knock-out mice is not feasible for AP2α, β and γ as the mice are not viable, which is different for AP2ε. The expression of the isoform AP2ε has been detected in the developing olfactory bulb, neural tissue, and by our group in cartilage, chondrocytes, and chondrosarcoma cells. In cartilage development, AP2ε plays a crucial role during late chondrocytic differentiation [18]. Furthermore, the loss of AP2ε in vivo leads to an enhanced osteoarthritis disease progression, indicating its role in pathological processes [19]. In melanoma, the role of AP2ε during progression has not been extensively examined and, therefore, is not fully elucidated yet. Understanding the function of AP2ε and regulatory networks in melanoma may provide insights into the underlying mechanisms of melanoma progression and potentially identify new therapeutic targets for this aggressive form of skin cancer.

## Materials and Methods

### Animals and animal models

Animals were kept under standardized conditions at a temperature of 20–22 °C, a relative humidity of 46-48% and a 12 h light–dark cycle. Mice were housed in groups of 2-5 per cage and had *ad libitum* access to water and food. For this study, an AP2ε knockout mouse strain (AP2ε^-/-^) [19] and Tg(GRM1)EPv mice [20], both on a C57Bl/6 background were used. Double transgenic mice were generated by crossbreeding of Tg(GRM1)EPv mice and AP2ε ^-/-^ mice, generating an AP2ε^-/-^/Tg(GRM1) strain. Tg(GRM1)EPv mice carry the metabotropic glutamate receptor 1 (GRM1) under the control of the melanocyte-specific dopachrome tautomerase (Dct, Trp2) promoter. Overexpression of GRM1 induces melanocytic hyperproliferation, leading to spontaneous development of pigmented cutaneous melanomas at hairless skin regions, with a short latency and 100% penetrance [20]. Starting with an age of 60 days, 9 AP2ε^-/-^/Tg(GRM1) and 9 Tg(GRM1) mice were monitored once a week for the development of melanocytic lesions and melanoma growth. After onset, the tumors were further graded for 9 weeks, as established and described previously [21, 22]. Tissue samples were collected, fixed in a solution of 4% paraformaldehyde/1xPBS embedded in paraffin or were immediately snap-frozen and stored at − 80 °C for further analysis. Breeding of animals was reported to the Ethics Committee of the Government of Middle Franconia according to §11 of the German Animal Welfare Act (RUF-55.2.2-2532-2-534-8). For routine genotyping, genomic DNA was prepared and analyzed by PCR as described elsewhere [19].

### Isolation and cultivation of murine and human melanoma cells

To generate primary melanoma cell lines, AP2ε^-/-^/Tg(GRM1) and age-matched Tg(GRM1) were sacrificed by cervical dislocation and lungs were dissected immediately. The tissue was briefly washed with 1x PBS. Subsequently, the lung tissue was cut into small pieces and was added to a mixture of 1 ml Dulbecco’s modified Eagle medium (DMEM, Sigma-Aldrich, München, Germany) and collagenase VIII from Clostridium histolyticum (Sigma-Aldrich, München, Germany). After incubating at 37 °C for 3 h the cell suspension was seeded into T25 flasks (Corning Inc., Corning, NY, USA). The cells were cultured in high-glucose DMEM supplemented with 10% FCS, 1% penicillin/streptomycin and 0.5 µg/mL amphotericin B (Sigma Aldrich, St. Louis, MO, USA) at 37 °C in humidified 8% CO_2_. To confirm that the isolated cells are from melanocytic origin, GRM1 expression was verified by RT-PCR, as previously described [23] (Supplementary Figure S1). Human melanoma cell lines Mel Juso, 501Mel, and Mel Im (FUCCI) were cultivated as indicated elsewhere [24, 25]. Mycoplasma contamination was regularly excluded for all primary cells and cell lines. Cell lines were authenticated using the short tandem repeat (STR) method.

### FUCCI Reporter Cells

The FUCCI (fluorescence ubiquitination-based cell cycle indicator)-labelled Mel Im cells were generated and quantified as described previously [24].

### Bioprinting

Spheroids for 3D printing were grown in a hanging drop assay as described previously [26]. Bioprinting was performed as described before in detail [27]. Briefly, cells or spheroids were harvested, and suspensions were mixed 1:11 with Cellink Bioink (CIB) (BICO Group, Gothenburg, Sweden) or ice-cold Matrigel (MG) (Corning Inc., Corning, NY, USA) to a final concentration of 3 ×10^4^ cells/ml or 160 spheroids/ml, respectively. The MG bioink was pre-gelled at room temperature for 30 min. For both materials, three-layered grid structures (1 cm^2^) were printed into 6-well plates. CIB constructs were crosslinked with 50 mM CaCl_2_, for five minutes, whereas MG constructs were polymerized by a temperature shift to 37 °C, for 30 min. Hardened constructs were cultivated in the respective cell culture medium at 37 °C and 8% CO_2_.

### Live cell imaging

To analyze the migratory behavior of Mel Im FUCCI in Cellink Bioink, printed constructs were glued to the well plate with Matrigel and covered with a culture medium. Constructs were kept in a cellVivo incubator chamber for the Olympus IX83 fluorescence microscope under their usual culture conditions over 8 days and pictures were taken automatically every 45 min.

### Luciferase assay

The activity of AP2 in human Mel Im or murine primary cell lines AP2ε^-/-^/Tg(GRM1) and Tg(GRM1) was determined using luciferase (LUC) assays. The respective cell lines were seeded into 6-well plates in duplicates and were transiently transfected with plasmid DNA containing the AP2-responsive element (underlined) on a pGL3Promoter-Plasmid or the empty vector ((GCCATGACTCATAAGGCCCTGGGCGGTCACTTTAAGCCATGACTCATAAGGCCCTGGGCGGTCACTTTAAGCCATGACTCATAAGGCCCTGGGCGGTCACTTTAA), Geneart, ThermoFisher, Waltham, USA) and co-transfected with a pRL-TK control vector (Promega Corp., Madison, WI, USA) using Lipofectamine LTX/Plus reagent according to the manufacturer’s instructions. Cells were harvested 24 h after transfection and printed in CIB or MG. After another incubation for 48 h, the constructs were lysed and the firefly LUC activity was quantified by a luminometric assay (Promega Corp., Madison, WI, USA). The data were normalized to renilla LUC activity encoded on the pRL-TK. For LUC assays of Mel Im cells after TFAP2E knockdown, cells were seeded in duplicates into 6-well plates and transfected with a control siRNA or siRNA against TFAP2E (Targetsequence: AAGGATGCCAAGCATCGGAAA) (QIAGEN) using the RNAiMAX Transfection Reagent (Invitrogen, Waltham, MA, USA) 24 h before transfection with the LUC-plasmids.

### Functional in vitro assays

Proliferation, attachment, migratory activity and spheroid growth of cells were analyzed in different functional assays as described in previous studies and in detail in Supplementary Materials and Methods [21, 22, 28].

### Transient transfection

Using the Lipofectamine LTX/Plus reagent (Invitrogen) Mel Juso or Mel Im cells were transfected with pCMX-PL2-TFAP2e plasmid containing the AP2ε coding sequence in a pCMX-PL2 backbone (kindly provided by Markus Moser) or the corresponding pCMX control vector plasmids according to the manufacturer’s instructions. Total RNA was isolated, or cells were used for further XTT cell viability, Clonogenic or Boyden chamber assays.

### Analysis of mRNA expression using real-time PCR

RNA isolation from human and murine melanoma cells was achieved using the E.Z.N.A.^®^ Total RNA Kit (Omega Bio-Tek, Norcross, GA, USA), RNA from bioprinted constructs was extracted using Trizol® Reagent, each according to manufacturer’s instructions. The concentration and purity of the obtained RNA were measured using the NanoDrop device (Peqlab Biotechnologie GmbH). Generation of cDNA was performed as previously described [29]. For real-time PCR, LightCycler® 480 II devices (Roche, Basel, Switzerland) were used with forward and reverse primers from Sigma-Aldrich (Supplementary Table 1).

### Western Blot Protein analysis

Total protein isolation of human melanoma cells, protein quantification and Western blot analysis were performed as described previously [29]. For detection of AP2ε protein, a custom-made, specific rabbit AP2ε-antiserum (1:5,000 in 5% BSA, Anamar Medical AB, Lund, Sweden) was used. Primary antibodies against MITF (1:500 in 5% BSA, Santa Cruz, sc-515925, RRID:AB_2828036), BRN2 (1:500 in 5% BSA/1x TBS-T, Santa Cruz, sc-393324, RRID:AB_2737347), Snail1 (1:1,000 in 5% BSA/1x TBS-T, Cell Signaling Technology Cat# 3879, RRID:AB_2255011) and E-cadherin (1:1,000 in 5% BSA/1x TBS-T, Cell Signaling Technology Cat# 3195, RRID:AB_2291471) were incubated overnight at 4 °C. A primary antibody against β-Actin (1:5,000 in 5% BSA, Sigma-Aldrich Cat# A5441, RRID:AB_476744) served as loading control. Horseradish peroxidase-conjugated secondary antibodies (HRP, Cell Signaling Technology Cat# 7074, RRID:AB_2099233 and RRID:AB_330924) were applied and a Chemostar chemiluminescence imager (Intas, Goettingen, Germany) was used for signal detection. The LabImage software (Version 4.2.3, Kapelan Bio-Imaging GmbH, Germany) was used for quantification.

### Immunohistochemistry

For immunohistochemical analysis of GRM1 on murine paraffin-embedded tissue samples, an anti-GRM1 primary antibody (SA-610-0200, 1:50, ENZO life Sciences, anti-rabbit) was used on 5 µm tissue sections as described previously [23]. To investigate the metastatic behavior, three sections of the lung of three animals were investigated. At least 10 fields per view per section of the animals were analyzed by two independent researchers. AP2ε expression in melanoma tissue from primary tumor or metastatic origin, in CIB or MG cultures was detected using a specific anti-AP2ε antibody by Anamar (1:20, rabbit) used in a previous study [18]. Sampling and handling of patient material was carried out in accordance with the ethical principles of the Declaration of Helsinki. The use of human tissue material had been approved by the local ethics committee of the University of Regensburg (application numbers 09/11 and 03/151). For quantification of AP2ε-positive cells, 10 fields of view per slide and 5 patient samples of primary tumor and metastasis, respectively, were counted. For immunohistochemical staining of MITF and BRN2-protein expression in CIB cultures, an anti-MITF primary antibody (1:500, Abcam Cat# ab233928, RRID:AB_2943147) and anti-BRN2 primary antibody (1:200, Cell Signaling Technology Cat# 12137, RRID:AB_2797827), was used, respectively. BRN2 and MITF staining was described quantitatively using “none”, “weak” or “strong” staining.

### Immunofluorescence

Immunofluorescence stainings were performed on 5 µm patient-derived human cutaneous malignant melanoma tissue. Briefly, Antigen retrieval was carried out with Citrate-Buffer pH 6.7 for 20 min at 99 °C before sections were permeabilized with 1% Triton X-100 in PBS and blocked with 1% BSA/PBS. Primary antibody was added and incubated overnight at 4 °C. Afterwards sections were incubated with the respective secondary antibody 1:400 in PBS containing 0.5% Triton X-100. The antibodies used are listed in Supplementary Table 2. In the final step, nuclei were stained with DAPI (1:10,000 in 1% BSA/PBS, Sigma Aldrich), and sections were mounted on coverslips with Aqua Polymount (Polysciences). An Olympus IX83 inverted microscope was used for the analysis of the immunofluorescent staining.

### RNA-Sequencing and bioinformatic analyses

RNA-Seq Library Preparation, data preprocessing, and analysis were performed as previously described [30]. Patient survival rates according to AP2α, AP2γ and AP2ε expression were analyzed by applying the TCGA-derived datasets published by the Protein Atlas Database (https://www.proteinatlas.org/ENSG00000137203-TFAP2A/pathology/melanoma), https://www.proteinatlas.org/ENSG00000087510-TFAP2C/pathology/melanoma, https://www.proteinatlas.org/ENSG00000116819-TFAP2E/pathology/melanoma. OncoDB (https://oncodb.org/index.html) was used for expression analysis of AP2α, AP2γ and AP2ε in human malignant melanoma tissue (MM), compared to normal skin tissue (non-tumor).

SRA files of the study La et al. [31] were downloaded from the Gene Expression Omnibus (GEO) database under the accession code GSE174520 and converted into Fastq files using sratoolkit (3.0.7). Read quality was assessed utilizing FastQC (v.0.12.1) before performing adapter trimming and quality filtering with fastp (0.23.4). Reads were mapped against the reference genome using the STAR (2.7.11a) aligner. The required STAR index was built based on the hg38 genome and GENCODE human transcript annotation (version 44). Sorted BAM files were indexed with samtools (1.17) index and mapping statistics were obtained via samtools flagstat. An RNA-based count matrix was generated using featureCounts (2.0.6) prior to running a differential gene expression analysis following the DESeq2 pipeline in R (4.3.1) and setting an adjusted p-value cutoff of < 0.1. Significantly up-regulated genes in quiescent cells were subsequently examined for potential AP-2 binding sites in the promoter region. Therefore, promoter regions which were defined from 1.0 kb upstream to 0.5 kb downstream of the corresponding annotated gene start (GENCODE annotation) were extracted using a custom R script. These sequences were then screened for an AP2 consensus sequence that was taken from the HOMER ChIP-seq database, utilizing the annotatePeaks.pl program within the HOMER (v4.10) software toolbox. The AP2 sequence logo was imported into R using the seqLogo package and the remaining plots were generated with ggplot2.

### Statistical analysis

Analysis and visualization of experimental results was done using GraphPad Prism 9 software (GraphPad Software Inc., San Diego, CA, USA). If not otherwise stated, at least 3 biological replicates were measured. All results are normalized to the respective control treatment and shown as mean ± SEM, if not otherwise stated. A critical value of *p < 0.05 was considered statistically significant.

## Results

### Analyses of AP2 family expression

**Fig. 1:**
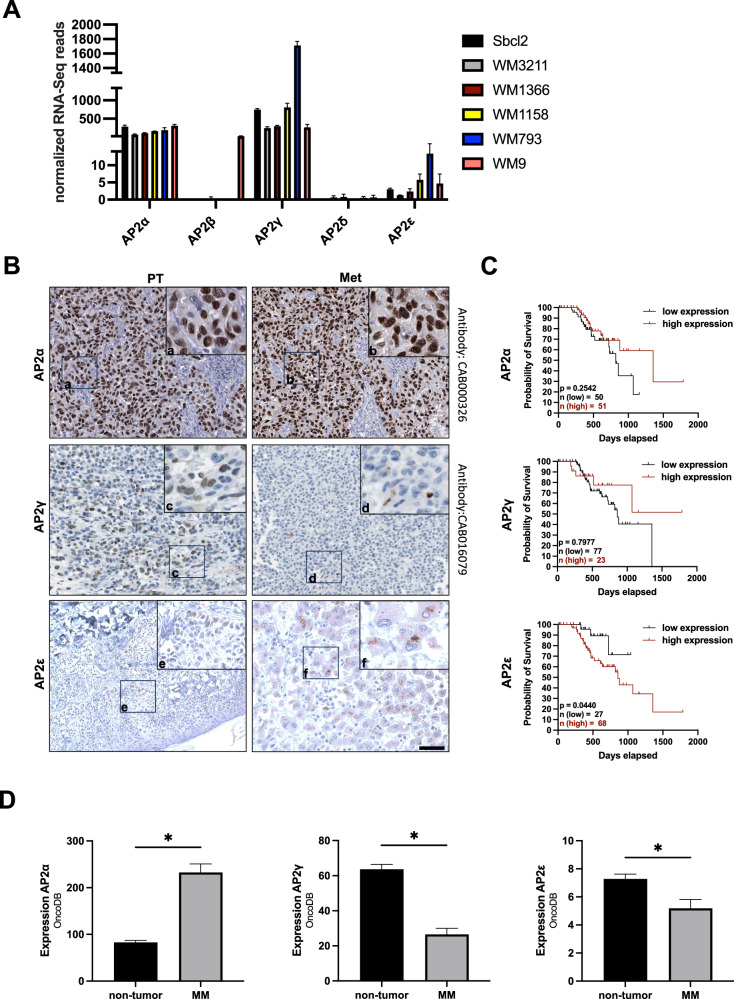
AP2-family expression pattern shows heterogeneity in melanoma cell lines. **A** RNA-Sequencing of different melanoma cell lines (SbCl2, WM3211, WM1158, WM793, WM9) depicting a heterogenic expression pattern of the 5 AP2-isoforms. RNA-Seq read counts were normalized to library size. **B** Immunohistochemical stainings of primary melanoma tumor (PT) or metastasis (Met). AP2α and AP2γ stainings are published by the human protein atlas (AP2α: https://www.proteinatlas.org/ENSG00000137203-TFAP2A/pathology/melanoma AP2γ: https://www.proteinatlas.org/ENSG00000087510-TFAP2C/pathology/melanoma; accessed on 08/09/2023, 11:51 am). AP2ε was stained with a specific anti-AP2ε-antiserum, established in our group [19]. a, b Image sections depicting high nuclear staining of AP2α in primary tumor and metastasis. c, d Image sections showing weak nuclear staining of AP2γ in primary tumor and low staining in metastasis. e, f Image sections depicting low nuclear staining in primary tumor and only low staining in approximately 5% of the cells in the metastasis for AP2ε. (Scale bar: 200 μm). **C** Kaplan-Meier survival curve analysis for AP2α, AP2γ, AP2ε in malignant melanoma was performed using the TCGA-derived datasets deposited on the ProteinAtlas database. Survival analysis was performed computationally applying log-rank testing. **D** Expression analysis for AP2α, AP2γ, and AP2ε published on OncoDB (https://oncodb.org/index.html) comparing normal skin tissue (non-tumor) and malignant melanoma (MM) tissue. Data information: Data are represented as mean ± SEM; *p < 0.05 (Two-tailed Student’s t-test).

Investigating RNA expression data of 2D-grown human melanoma cell lines (SBcl2, WM3211, WM1366, WM1158, WM793, WM9) revealed a heterogenic expression pattern of the AP2 family members (Fig. 1A). Generally, AP2δ showed a low to no expression in all melanoma cell lines and AP2β was only expressed in the cell line WM9. In contrast, AP2α, AP2γ and AP2ε were expressed in all tested cell lines, with the highest expression levels of isoform AP2γ, followed by AP2α and AP2ε. To define the in vivo situation, we evaluated available immunohistochemistry of melanoma sections from “*The Human Protein Atlas”* (ProteinAtlas) for AP2α, AP2γ and AP2ε [32]. Here, AP2α showed strong nuclear expression in most of the analyzed samples ( > 70%) (Fig. 1B, a, b). AP2γ appeared with only weak nuclear staining in the primary tumor and almost no staining in metastatic tissue (Fig. 1B, c, d). For higher specificity, we stained AP2ε with our own established anti-AP2ε-antiserum [18]. After quantification of AP2ε-positive cells (Supplementary Figure S2), we revealed approximately 3,5% cells with AP2ε expression in primary tumors. We also detected 5% of cells with high AP2ε expression in metastases (Fig. 1B, e, f). Additionally, we analyzed TCGA-derived patient data (applying the ProteinAtlas database) for survival probability. Patients with high AP2α or AP2γ expression showed a tendency to improved prognosis (Fig. 1C, first and second panel). Interestingly, high expression of AP2ε was significantly associated to worse prognosis (Fig. 1C, third panel). Additionally, expression analysis from OncoDB (https://oncodb.org/index.html) comparing normal skin tissue (non-tumor) with malignant melanoma tissue (MM) revealed elevated expression of AP2α, whereas AP2γ and AP2ε showed reduced expression in MM (Fig. 1D).

### Analysis of AP2 expression in 3D in vitro models

**Fig. 2:**
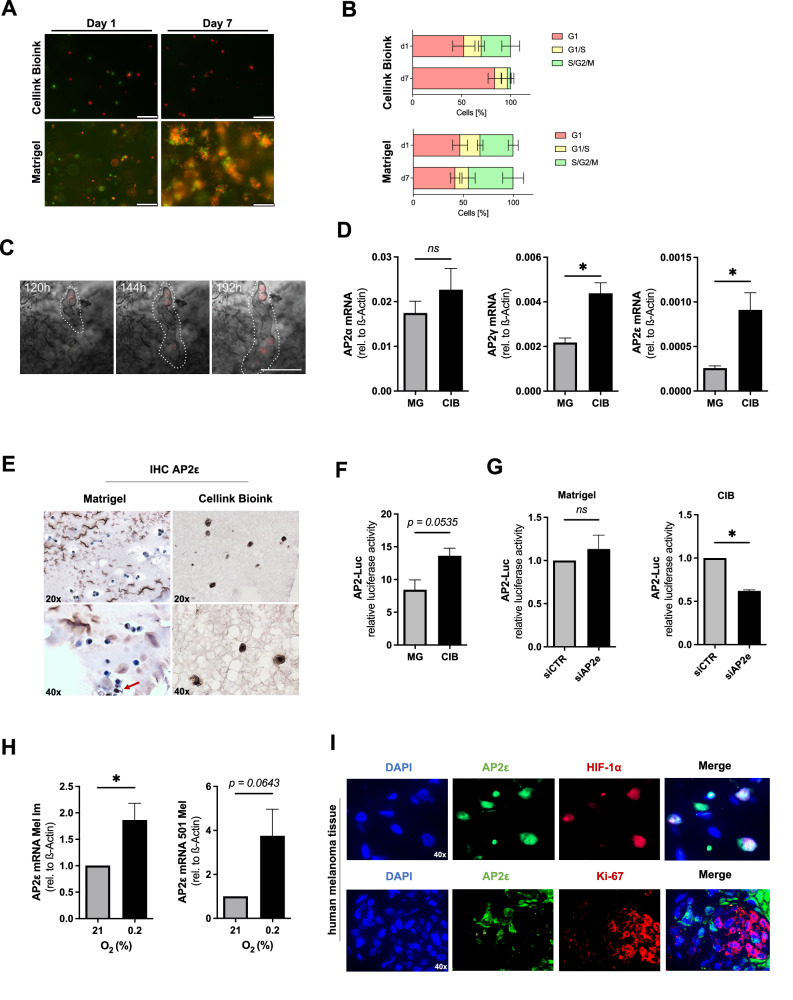
Melanoma cells reveal increased AP2ε expression and activity in quiescent cells in a biofabricated model. **A** Mel Im cell growth over 7 days (Scale bar = 200 µm). **B** Cell cycle analysis of Mel Im FUCCI in CIB and MG, respectively. **C** Mel Im migration in CIB during cultivation (Scale bar: 200 µm). **D** mRNA-expression analysis for AP2α, AP2γ, and AP2ε in MG and CIB (*n* = *3*). **E** Immunohistochemical staining for AP2ε in Mel Im cells bioprinted in CIB and MG. **F** Relative luciferase activity in Mel Im cells transfected with an AP2-Luciferase construct and afterwards cultivated in MG or CIB (*n* = *3*). **G** Relative luciferase activity in Mel Im cells transfected with an AP2-Luciferase construct after transfection of siRNA against AP2ε and control-siRNA, respectively. After transfection cells were cultivated in MG (*n* = *3*) or CIB (*n* = *4*). **H** mRNA-expression analysis for AP2ε after cultivating the melanoma cell lines Mel Im and 501 Mel under hypoxic conditions (*n* = *3*). **I** Immunofluorescence images of AP2ε and HIF1-α or Ki-67 in the human melanoma tissue samples (Magnification: 40x). Data information: All data from at least three independent experiments are represented as mean ± SEM; *p < 0.05; *ns*: not significant; (Two-tailed Student’s t-test) (**D**, **F**, **G**); (Scale bars (**A**, **C**, **E**, **I**): 10 µm).

To generate in vitro data that more closely resemble the in vivo situation, we used 3D-biofabricated models recently established in our group [27]. The human melanoma cell line Mel Im FUCCI was cultivated either in the basement-membrane like Matrigel (MG) matrix or in Cellink Bioink (CIB) consisting of alginate and nanofibrillar cellulose. Cultivating the cells in MG revealed strong proliferation, whereas the cells in CIB stayed in a G1 cell cycle arrest over 7 days in culture (Fig. 2A, B). The non-proliferative phenotype observed in the biofabricated model hints towards the induction of a quiescent phenotype, which is associated to stronger migratory potential. Accordingly, life-cell-imaging of Mel Im FUCCI in CIB culture revealed migratory activity (Fig. 2C). These stem-like cancer cells are speculated to be a small percentage in vivo, but when using the defined CIB culture, this phenotype accumulates. Next, we analyzed the expression of the AP2 isoforms α, γ and ε in the human melanoma cell line Mel Im in both the proliferative (embedded in MG) and quiescent cell state (embedded in CIB) (Fig. 2D). Interestingly, AP2α mRNA expression was not altered between the two states, whereas AP2γ and AP2ε mRNA expression was significantly increased in the quiescent cells.

The fact that patients with high AP2ε expression showed worsened survival prognosis and at the same time, only a small percentage of cells in melanoma metastasis were AP2ε positive, hinted towards a yet unknown crucial tumor-promoting role of cells expressing this isoform. We therefore performed immunohistochemistry of CIB encapsulated cells for AP2ε, revealing strong nuclear staining in all of the cells (Fig. 2E, left panel). Otherwise, only a small number of cells were stained positive in MG (Fig. 2E, right panel). Luciferase assays confirmed the increased activity of AP2 in the non-proliferating and migratory cell phenotype in CIB (Fig. 2F). Specifically silencing AP2ε using siRNA-technology revealed the increased relevance of this isoform in the accumulated quiescent cells, compared to the heterogeneous phenotype in MG (Fig. 2G). Next, we performed a bioinformatical analysis of an independent study, in which La and colleagues [31] compared gene expression profiles of quiescent and cycling melanoma cells. We first analyzed for significantly deregulated genes (DEGs) (Supplementary Figure S3A) and screened for an AP2-binding motif in the near proximal promotor region of significantly upregulated genes (Supplementary Figure S3B). Surprisingly, we revealed that 76% of the upregulated genes in quiescent cells have an AP2-binding motif in their promotor (Supplementary Figure S3C), further supporting the role of AP2 in inducing or maintaining a non-proliferative, quiescent cell phenotype. In a previous study, we investigated the regulation of AP2ε by Hypoxia-inducible factor-1 (HIF1-α) during chondrogenic differentiation of murine mesenchymal stem cells [33]. Since a quiescent phenotype is often triggered by hypoxia, we cultivated the melanoma cell line Mel Im and 501 Mel under reduced oxygen tension (0.2%) and observed an enhanced AP2ε gene expression (Fig. 2H). Additionally, immunofluorescence staining of human melanoma tissue showed an induction of the AP2ε protein expression in HIF1-α positive nuclei (Fig. 2I, upper panel). Supporting the non-proliferative quiescent phenotype, AP2ε expressing cells did not show an expression of Ki-67 in immunofluorescence staining on human melanoma tissue (Fig. 2I, lower panel).

### Analysis of the role of AP2ε in melanoma cell proliferation in vitro and in vivo

**Fig. 3:**
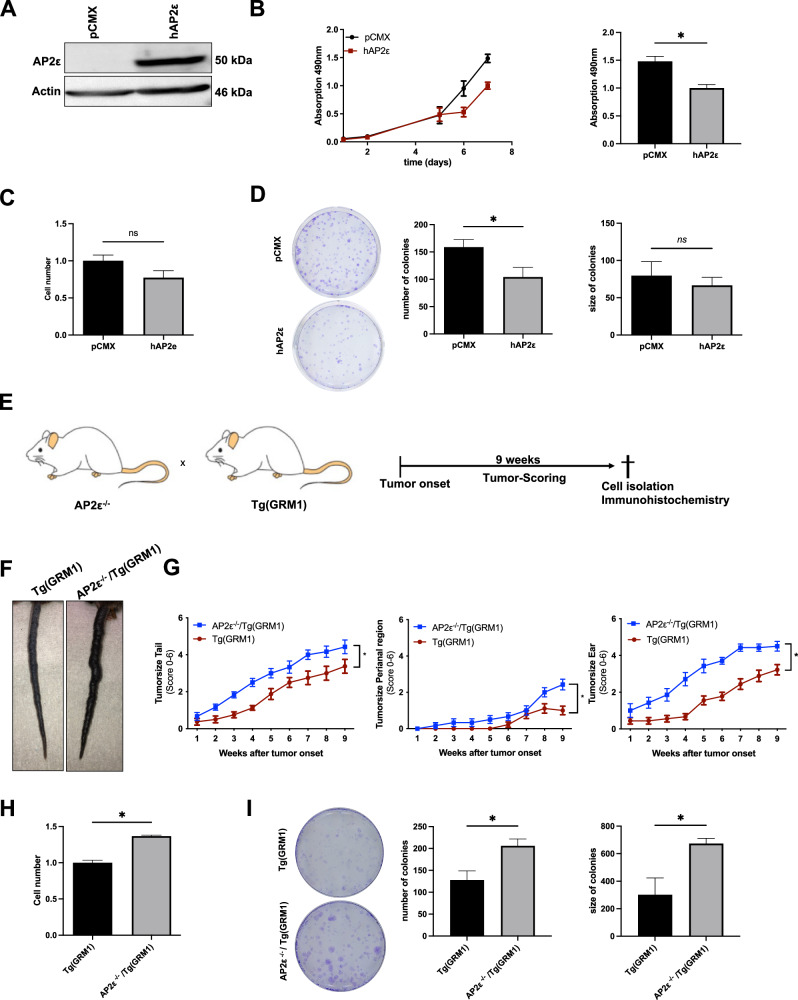
The loss of AP2ε leads to enhanced tumor progression in vivo. **A** Representative Western Blot images depicting human AP2ε protein levels after transfection with the overexpression (OE) plasmid and control vector pCMX (*n* = 3). β-Actin served as loading control. **B** XTT Cell viability analysis for 7 days after transfection with OE-plasmid and pCMX control vector (left panel). After seven days, absorption at 490 nm was significantly reduced (right panel) (*n* = 3). **C** Cell counting-assay revealed reduced proliferation (*n* = 3). **D** Colony number and colony size in two-dimensional clonogenic assays (*n* = 3); Data information: All data are represented as mean ± SEM; *p < 0.05 (two-tailed Student’s t-test) (**B**, **C**, **D**). **E** Crossing scheme and experimental scheme of tumor scoring paradigm: AP2ε^-/-^ mice were crossbred with Tg(GRM1) mice to generate an AP2ε^-/-^/Tg(GRM1) (tg/tg) genotype. Mice were monitored until tumor onset was observed. After tumor onset, mice were scored once a week for 9 weeks and afterwards sacrificed for tumor cell and tissue isolation. **F** Exemplary images of the different tumor growth on the tail of age matched Tg(GRM1) mice (left) and AP2ε^-/-^/Tg(GRM1) mice. **G** Staging of tumor growth intensity at the perianal region, tail, ear for 9 weeks after tumor onset. **H** Cell counting-assay revealed reduced proliferation (*n* = 3). **I** Colony number and colony size in two-dimensional clonogenic assays (*n* = 3). Data information: All data from at least three independent experiments are represented as mean ± SEM; *p < 0.05 (two-tailed Student’s t-test) (**B, C, D**). number of experimental animals for (**B**) Tg(GRM1) *n* = 9 and AP2ε^-/-^/Tg(GRM1) *n* = 9.

The impact of other AP2-Isoforms in cancer progression, such as AP2α, has already been determined in several studies [14, 34, 35]. Interestingly, we could only observe AP2ε expression in Ki-67 negative, non-proliferative cells in human melanoma tissue. To confirm the impact of AP2ε on cell proliferation and melanoma progression, we used the human melanoma cell line Mel Juso and revealed reduced proliferation after overexpression of AP2ε (Fig. 3A, B, C) and reduced colony growth in a clonogenic assay (Fig. 3D). To finally determine whether AP2ε has an impact on melanoma development and progression in vivo, we crossbred AP2ε-deficient mice with a transgenic melanoma model (Tg(GRM1)), a murine model for spontaneous melanoma development [20, 36]. Tumor progression was assessed for 9 weeks after tumor onset (Fig. 3E). Comparing the tumor progression of AP2ε^-/-^/Tg(GRM1) and Tg(GRM1)-mice we observed significant induction of local tumor progression for the tail (Fig. 3F, G first panel), perianal region (Fig. 3G, second panel), and ear (Fig. 3G, third panel) in the AP2ε deficient mice. We then generated cell lines of metastatic melanoma from the lung and characterized the cells using functional in vitro assays. Measuring proliferation by cell counting, we revealed enhanced proliferation of AP2ε^-/-^ cells compared to AP2ε-expressing cells (Fig. 3H). Additionally, the AP2ε^-/-^ cells showed significantly enhanced anchorage-dependent colony formation and colony size as compared with Tg(GRM1)-control cells (Fig. 3I). This stronger proliferation of AP2ε deficient cells goes along with an induction of AP2α expression (Supplementary Figure S4).

### Role of AP2ε in metastasis and quiescence

**Fig. 4:**
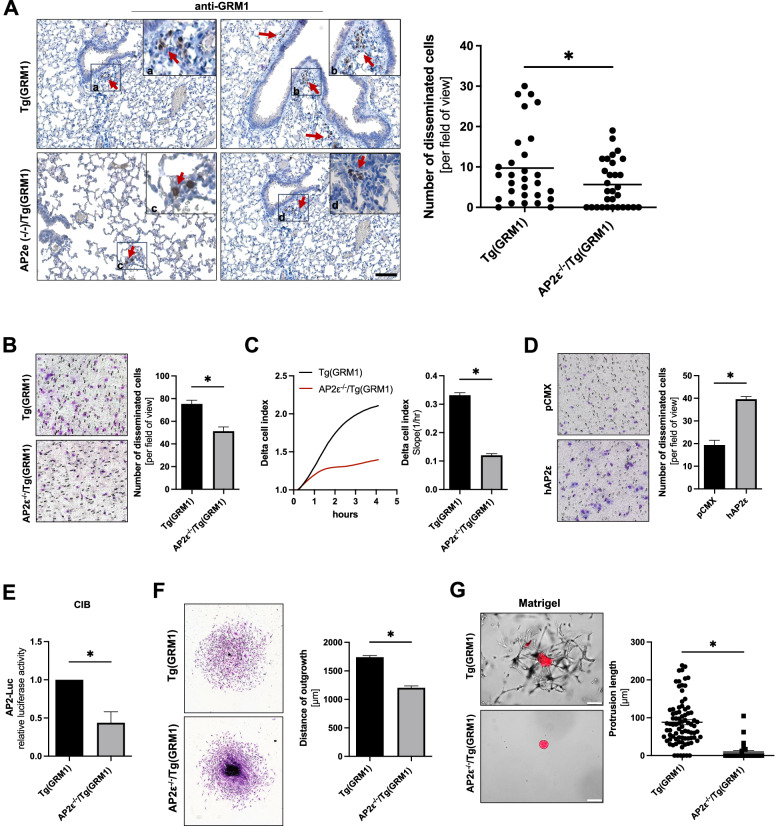
Reduced metastasis in AP2ε^-/-^/Tg(*GRM1*) mice. **A** Representative AP2ε immunohistochemical analysis of lung metastasis from Tg(GRM1) and AP2ε^-/-^/Tg(GRM1) mice. a, b, c, d Red arrows depicting AP2ε positive nuclear stainings. Number of disseminated cells per field of view of 3 mice per genotype in 10 sections per mice have been counted (*n* = 3). (Scale bar: 200 µm). **B** Migratory behavior of AP2ε^-/-^/Tg(GRM1) cells compared to Tg(GRM*1*) using the Boyden chamber model. (*n* = 3). **C** Attachment analyses by using the xCELLigence system of Tg(GRM1) and AP2ε^-/-^/Tg(GRM1) for cells from metastatic lung tissue (Delta Cell index = relative change in measured impedance to represent cell status) (*n* = 3). **D** Migratory behavior of Mel Im cells transfected with the AP2ε-overexpression plasmid compared to control vector pCMX using the Boyden chamber model. (*n* = 3). **E** Relative luciferase activity in primary AP2ε^-/-^/Tg(GRM1) and Tg(GRM1) cells, respectively, transfected with an AP2-Luciferase construct and afterwards cultivated in CIB (*n* = *3*). **F** Spheroid outgrowth assay depicting reduced migratory activity in AP2ε^-/-^/Tg(GRM1) spheroids. Quantification of the distance of outgrowth in µm. **G** Embedded AP2ε^-/-^/Tg(GRM1) and Tg(GRM1) spheroids after 14 days in MG. White arrows depicting outgrown cells from the Tg(GRM1) spheroid. Protrusion lengths of 9 spheroids per condition have been counted after 10 days in MG (*n* = 3). Data information: All data from at least three independent experiments are represented as mean ± SEM; *p < 0.05 (two-tailed Student’s t-test) (**B**, **C**, **D**, **E**); For **A** and **G** an unpaired t-test with Welch’s correction was applied; number of experimental animals (**A**) *n* = 3.

After we clearly demonstrated the inhibitory role of AP2ε in melanoma cell proliferation, we wanted to understand TCGA analysis, depicting a worse prognosis in patients with tumors expressing high amounts of AP2ε. As metastasis is the main cause of death for cancer patients, and since AP2ε is upregulated in the migratory phenotype, we next investigated the impact of AP2ε loss on metastasis in the Tg(GRM1) mouse model. Surprisingly, by performing immunohistochemistry of lung sections, we observed strongly reduced metastatic burden in the AP2ε deficient mice (Fig. 4A). This again supports the role of AP2ε in metastasis and a migratory-invasive behavior. Next, we isolated murine metastatic cell lines from the lungs and analyzed the migratory behavior in Boyden chamber migration assays (Fig. 4B). Here we could confirm the strong impact of AP2ε on the migratory potential of the tumor cells. We further analyzed attachment of these cells by using the xCELLigence system and also detected a strongly reduced cell attachment comparing AP2ε^-/-^/Tg(GRM1) and Tg(GRM1) cells (Fig. 4C). To further confirm the role of AP2ε in cell migration, we conducted Boyden chamber assays using the human melanoma cell line Mel Im overexpressing AP2ε and revealed a significant increase in migrating cells compared to the control cells (Fig. 4D). Performing Luciferase assays revealed reduced activity of the AP2-Luc construct in AP2ε^-/-^ cells cultured in CIB (Fig. 4E). To further support our findings, we generated spheroids to define the migratory potential by an outgrowth assay. In line with our data, the spheroid outgrowth assay also revealed a reduced migratory potential of the AP2ε deficient cells (Fig. 4F). Embedding the murine spheroids into MG, we observed significantly decreased invasion of AP2ε^-/-^ cells into the surrounding matrix, again underpinning the crucial role of AP2ε in the metastasis (Fig. 4G).

### AP2ε regulates phenotypic plasticity

**Fig. 5:**
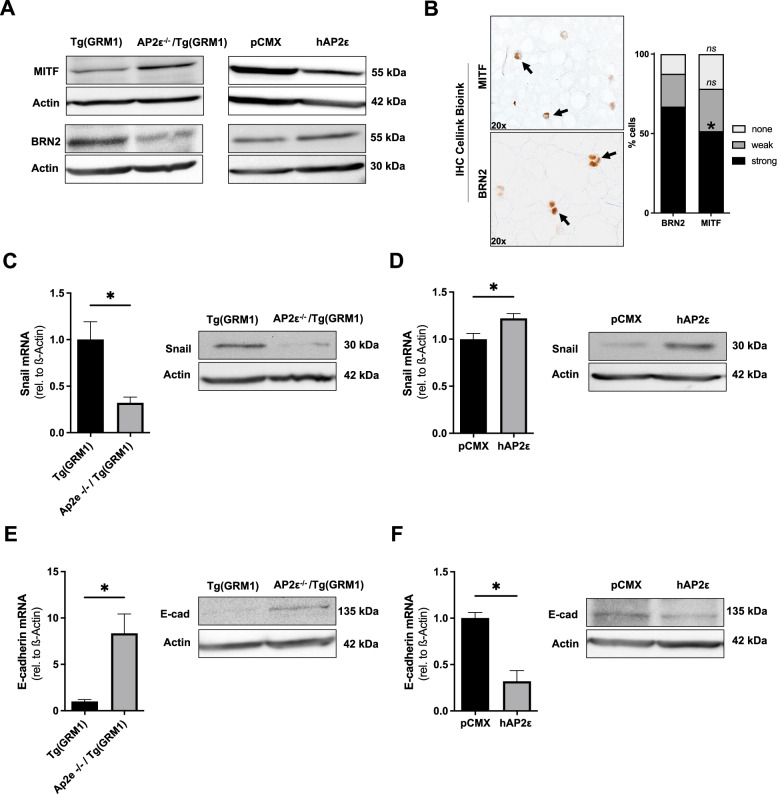
AP2ε regulates phenotypic plasticity of melanoma cells. **A** Representative Western Blot images depicting murine MITF and BRN2 protein levels in primary AP2ε^-/-^/Tg(GRM1) and Tg(GRM1) cells (left panels) and human MITF and BRN2 in Mel Juso cells transfected with the AP2ε-overexpression (hAP2ε) plasmid and control vector pCMX (right panels). β-Actin served as loading control. **B** Immunohistochemical staining of MITF and BRN2 protein expression in Mel Im melanoma cells bioprinted in CIB (Magnification: 20x). Quantification of percentage of cells with “no”, “weak”, or “strong” MITF and BRN2 expression. Data are represented as mean ± SEM; *p < 0.05; *ns*: not significant (Two-way-ANOVA with Fisher’s LSD test). **C** mRNA- and protein expression analysis for Snail1 in AP2ε^-/-^/Tg(GRM1) and Tg(GRM1) cells (*n* = 3). **D** mRNA- and protein expression analysis for Snail1 in Mel Juso cells transfected with the AP2ε-overexpression (hAP2ε) plasmid and control vector pCMX (*n* = 3). **E** mRNA- and protein expression analysis for E-cadherin in AP2ε^-/-^/Tg(GRM1) and Tg(GRM1) cells (*n* = 3). **F** mRNA- and protein expression analysis for E-cadherin in Mel Juso cells transfected with the AP2ε-overexpression (hAP2ε) plasmid and control vector pCMX (*n* = 3). Data information: All data from at least three independent experiments are represented as mean ± SEM; *p < 0.05 (Two-tailed Student’s t-test; *ns*: not significant).

Changing of expression of the Microphthalmia-associated Transcription Factor (MITF) and the POU domain transcription factor BRN2 (POU3F2) have already been linked to the metastatic mechanism of phenotype switching in malignant melanoma [37]. We, therefore, investigated protein expression of MITF and BRN2 in AP2ε^-/-^/Tg(GRM1) cells compared to Tg(GRM1) cells and observed a MITF^high^/BRN2^low^ phenotype, indicating a more proliferative cell state. To prove an AP2ε-dependend regulation of MITF and BRN2 expression, we further investigated the protein expression of these proteins in Mel Juso, overexpressing AP2ε. In line with our data, we could detect a MITF^low^/BRN2^high^ phenotype (Fig. 5A). Next, we investigated the MITF and BRN2 protein levels in quiescent melanoma cells printed in CIB. Here we could observe significantly less MITF^high^ than BRN2^high^ expressing cells, supporting a non-proliferative but migratory phenotype in CIB (Fig. 5B). Since the cells seem to gain mesenchymal-like properties due to an enhanced expression of AP2ε, we also investigated the process of epithelial-mesenchymal-transition (EMT). The transcription factor Snail1 is directly involved in the phenotypic switch from a proliferative to a mesenchymal-like migrating cellular state [38]. Interestingly, we could detect an AP2ε-dependent regulation of Snail1. The mRNA expression and protein level of Snail1 was significantly reduced in AP2ε^-/-^/Tg(GRM1) cells and significantly enhanced in Mel Juso transfected with the AP2ε overexpression plasmid compared to control (Fig. 5C, D). Snail1 directly suppresses the expression of the cell-adhesion glycoprotein E-cadherin (CDH1), leading in turn to the loss of intercellular junctions, and therefore promoting cellular migration and metastasis [39]. Supporting a reduced migratory phenotype and reduced metastasis in AP2ε^-/-^/Tg(GRM1) mice, we observed enhanced expression of E-cadherin in AP2ε^-/-^/Tg(GRM1) cells and reduced expression of E-cadherin in AP2ε-overexpression cells (Fig. 5E, F).

## Discussion

In this study, we investigated the complex role of the AP2 transcription factor family, with focus on AP2ε, in human melanoma. The family of AP2 transcription factors has already been attributed to melanoma progression in the past [5, 13, 40, 41]; however, a clear role of the different family members was not defined until today. In our study, we point out that AP2ε might play a key role in melanoma metastasis. Melanoma cells exhibit phenotypic plasticity, transitioning between differentiated (MITF^high^/BRN2^low^) and invasive (BRN2^high^/MITF^low^) states [42, 43]. This molecular plasticity is associated with increased tumor aggressiveness, resistance to therapies, and metastasis. AP2ε is, based on our data, implicated in the phenotypic plasticity of melanoma cells by regulating the transition between these two states. Changes in AP2ε protein expression lead to a distinct MITF/BRN2 phenotype. An AP2ε-deficiency resulted in a MITF^high^/BRN2^low^, higher levels of AP2ε in a MITF^low^/BRN2^high^ protein pattern. Additionally, we could detect a MITF^low^/BRN2^high^ protein pattern in quiescent melanoma cells printed in CIB, in which we could already prove an induction of AP2ε expression. Summarizing, these data show that AP2ε is an important player in the regulation of the MITF/BRN2 switch. Supported by our in vitro and in vivo findings, high AP2ε expression is associated with a highly invasive, metastatic phenotype in melanoma, most impressively demonstrated by the strongly reduced metastases in the AP2ε-deficient melanoma mouse model. This impact of AP2ε on the migratory phenotype is supported by other recent findings, demonstrating that AP2ε regulates the expression of genes involved in melanoma invasion and metastasis, like matrix metalloproteinases (MMP13) in non-melanoma cells [19]. Further studies also link AP2ε to a highly migratory and low proliferative phenotype in different species. Hong et al. revealed that in Xenopus, AP2ε expression persists in a subset of migrating cranial NC cells important to populate the pharyngeal arches [44]. Lin et al. could show that proliferating, non-migratory cells in basal vomeronasal sensory neuron development lack AP2ε expression, whereas migratory cells show higher expression [45]. Our biofabricated MG model nicely resembles the cancer cell phenotype of human tumor tissue in vivo within a heterogenic primary lesion, in which the population of cells with a high AP2ε expression might be more inclined to metastasize. This is in line with the fact that AP2ε-deficient cells are no longer able to evade from MG-embedded tumor spheroids. In a recent study, Kenny et al. observed strongest AP2ε expression in melanoblasts analyzing sorted MITF-positive cells in zebrafish embryos 28 h after fertilization, revealing that melanoblasts, derived from neural crest cells, express high levels of AP2ε during migration, supporting our findings [46].

In our study, we could further demonstrate that AP2ε not only plays an important role in melanoma metastasis but also in cellular plasticity. Specific effects of AP2ε on melanoma plasticity are supported by additional studies in zebrafish, specifically showing the impact of AP2ε on EMT of neural crest cells [47] or in mice, demonstrating effects on the cellular plasticity of mature apical vomeronasal sensory neurons [48]. In this study, we could reveal the regulation of the EMT-associated protein Snail1 by AP2ε. Snail1 is known to be an important inducer of EMT, with a clinical correlation of higher Snail1 expression and poor prognosis [49]. Snail directly represses the expression of the extracellular glycoprotein E-cadherin, by binding to its promoter in melanoma cells [50]. The downregulation of the tumor suppressor protein E-cadherin and, therefore, the loss of cell–cell adhesion and cell junctions is associated with EMT, which occurs during the process of metastasis [51]. In line with this data, the AP2ε-deficient cells show a low Snail1 expression, therefore a high E-cadherin expression, which led to a reduced metastatic load in the AP2ε-deficient mice.

We speculate that the expression level of AP2ε is a key player in determining this plasticity. We showed that AP2ε expression in general is significantly reduced in malignant melanoma tissue compared to normal skin tissue. With this present study, we could determine that a reduced expression of AP2ε is associated with enhanced proliferation of the tumor cells. In the context of a fully developed heterogenic primary melanoma, we believe that the upregulation of AP2ε in only a small number of cells within this lesion is crucial for the worse survival probability of the patients, as it mediates increased migratory capacity of these single cells leading to the formation of metastases. Tellez et al. linked a more malignant melanoma phenotype to a decrease in AP2α expression by regulating PAR1 [52]. We suggest, that this decrease is paired with the increase of AP2ε expression, leading to a specific ratio between these two paralogs. A study of Seberg et al. further supports this idea of a ratio between the family members being involved in AP2 member-specific regulation, revealing that in a zebrafish model AP2 family members AP2α and AP2β impact melanocyte differentiation and potentially the phenotype of melanoma cells [10]. This is supported by our data revealing an induction of AP2α expression in the murine melanoma tissue after loss of AP2ε. The interplay of the isoforms, also not understood today, is generally accepted, as summarized in a recent review [53]. However, studies addressing this aspect are mainly feasible in vivo, like in our study. This is seen in Liu et al., were differences between AP2α and β could not be defined although these were observed e.g. in the Seberg in vivo study [10, 54].

Based on the phenotype-switching model from Hoek et al., the AP2ε-positive cells could also be classified as quiescent [43]. Quiescent cells are characterized by a G1 cell-cycle arrest and induced tumor dormancy, which might make them resistant to chemotherapy and more likely to be invasive [55]. The biofabricated approach with CIB specifically allowed us to induce the quiescent G1-arrest and to address the role of AP2ε in this specific cell phenotype. The induction of AP2ε in the defined quiescent cells seems to counteract AP2α specific effects on proliferation.

Although, we did not address the question whether the quiescent phenotype of the AP2ε-positive cells in vitro and in vivo resembles stem cell-like cells, recent studies support this hypothesis. ChIP Enrichment Analysis (ChEA) predicted upstream transcription factors of *TFAP2Ε* expression are amongst other transcription factors, such as JARID1B/KDM5B, Nanog, Sox10 and Sox2, associated with induction of stemness in melanoma (https://maayanlab.cloud/archs4/gene/TFAP2E#tissueexpression) [56, 57]. Interestingly, JARID1B was also shown to be required for metastatic progression. In a study of Xia et al. HIF expression was shown to be an important factor for the dynamic regulation of JARID1B in HepG2 hepatocellular carcinoma and U87 glioblastoma-astrocytoma cells [58], further supported by Roesch et al. for melanoma [56]. The study by Kenny et al. revealed strong AP2ε expression in stem-cell-like melanoblasts in zebrafish embryos [46]. Further, we demonstrated the regulation of *TFAP2E* by HIF in this present study in malignant melanoma and a previous study in cartilage, which also links AP2ε expression to stem-cell-like features [33]. Hypoxia and HIF1 are generally known to be important in cancer stem cell development [59], inducing, for example, the expression of OCT4, SOX2, NANOG and Krüppel-like factor 4 (KLF4) as stem cell markers [60, 61]. Interestingly, Steunou et al. could directly link the overexpression of HIF1α in melanoma cells to increased cell migration [62], fitting our observation of HIF1α and AP2ε co-expression or complete absence in a subpopulation of the tumor cells.

In summary, understanding the role of AP2ε provides new insights into the plasticity and migratory potential of melanoma cells. Therapeutic strategies targeting AP2ε could aim to restore or stabilize the proliferative state (AP2ε^low^) while suppressing the quiescent, invasive state (AP2ε^high^), potentially limiting tumor progression and metastasis. Targeting AP2ε in an early disease state could prevent the formation of metastasis by reducing migratory potential, however, leading to a more proliferative cell state, probably making the cells more sensitive for chemotherapeutics. Further research is needed to uncover the precise mechanisms and signaling pathways involved in this new regulatory network and its therapeutic implications for melanoma treatment.

### Supplementary information

Original Data

Suplementary material and methods

Supplementary Figures

## Data Availability

The RNA-sequencing data used in this study have been deposited in the NCBI BioProject database (https://www.ncbi.nlm.nih.gov/bioproject/) and can be accessed with the BioProject accession number PRJNA839865.
