# Supplementary material for: Transcription factor activating enhancer-binding protein 2ε (AP2ε) modulates phenotypic plasticity and progression of malignant melanoma
Source: Cell Death Dis. 2024 May 21;15(5):351. doi: 10.1038/s41419-024-06733-3 (PMC11109141; doi:10.1038/s41419-024-06733-3)

Original Data

Figure 3 A – Western Blot

AP2ε

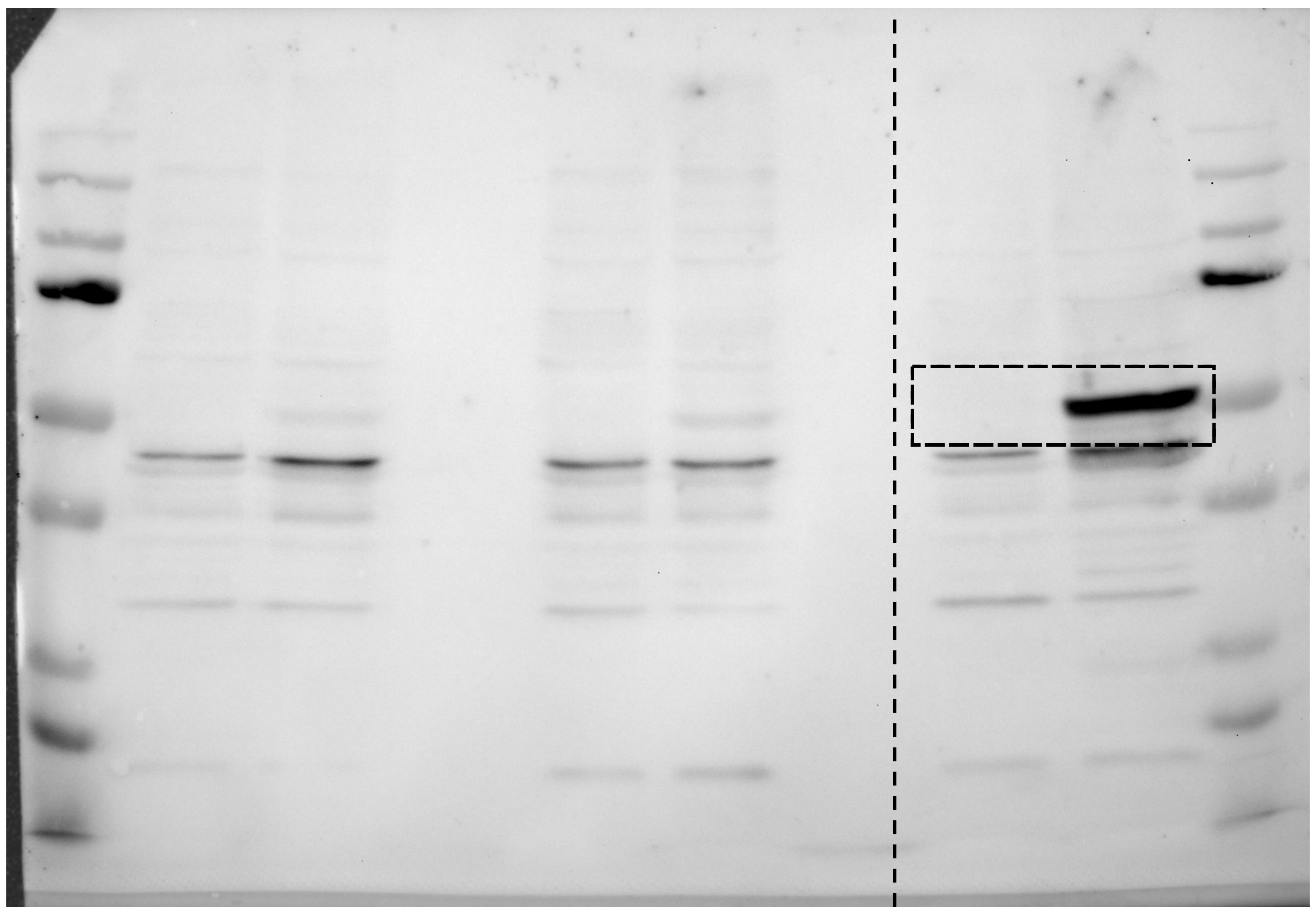

β-actin

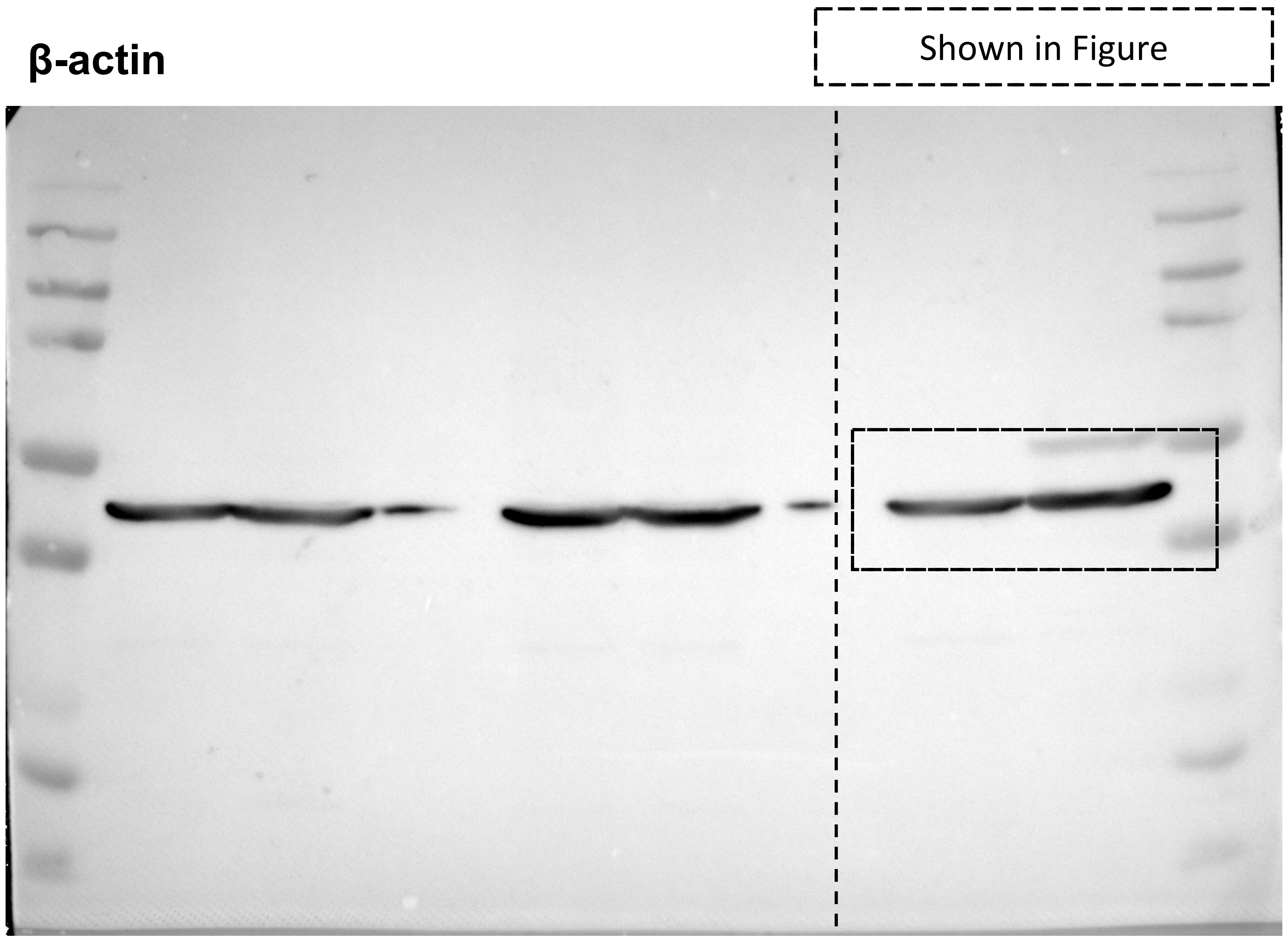

Ponceau S

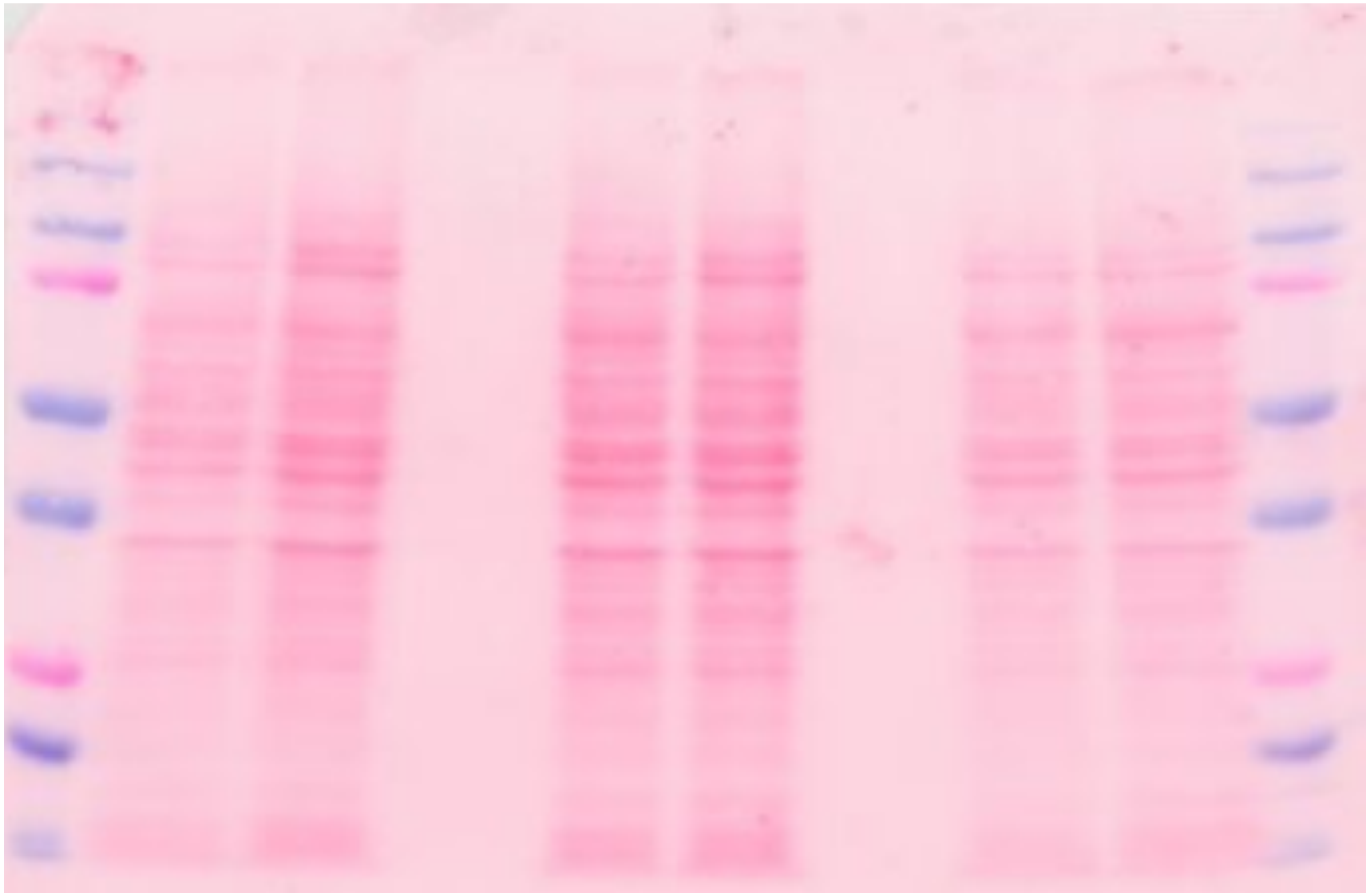

Figure 3 D – Clonogenic Assay

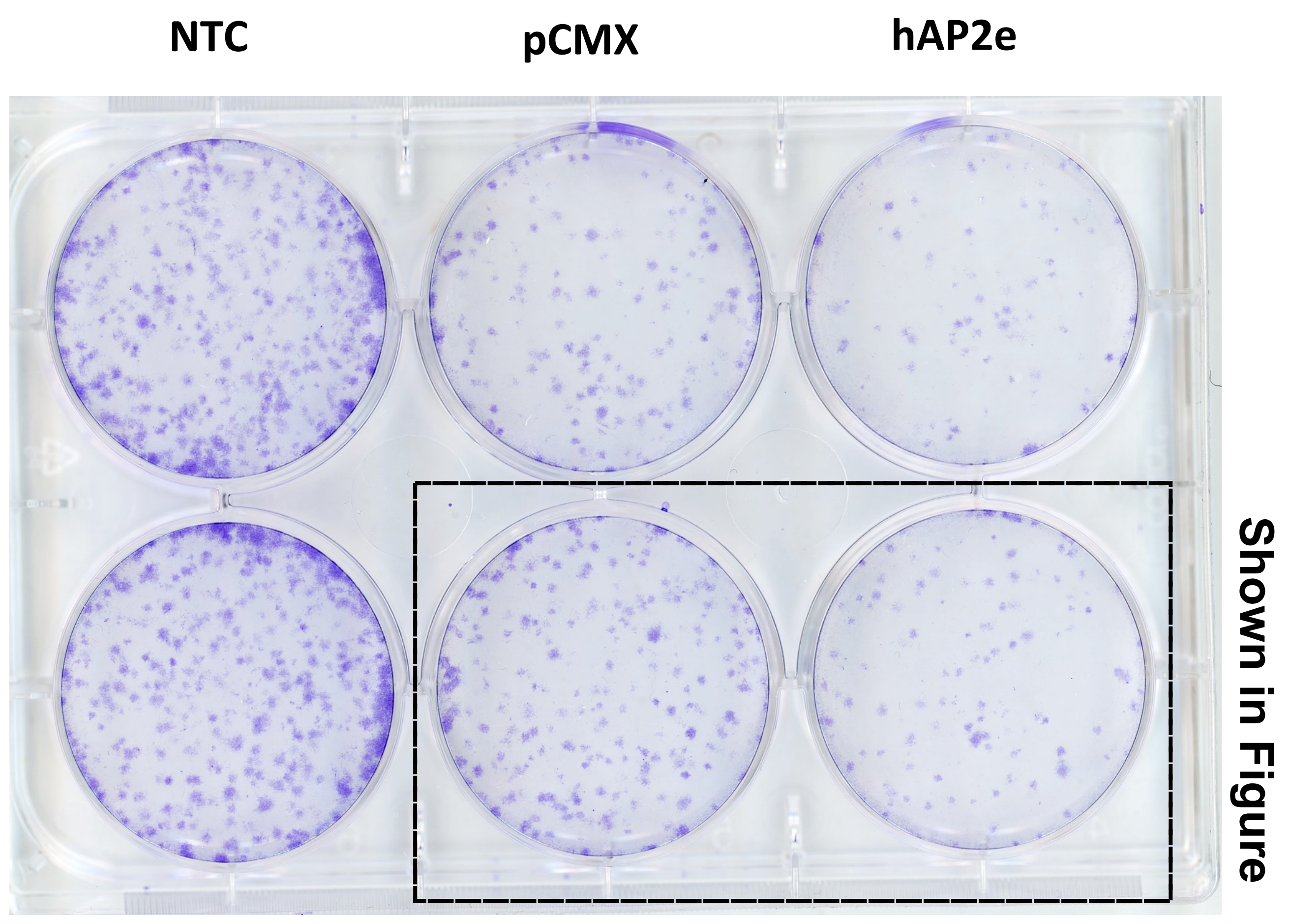

Figure 3 I – Clonogenic Assay

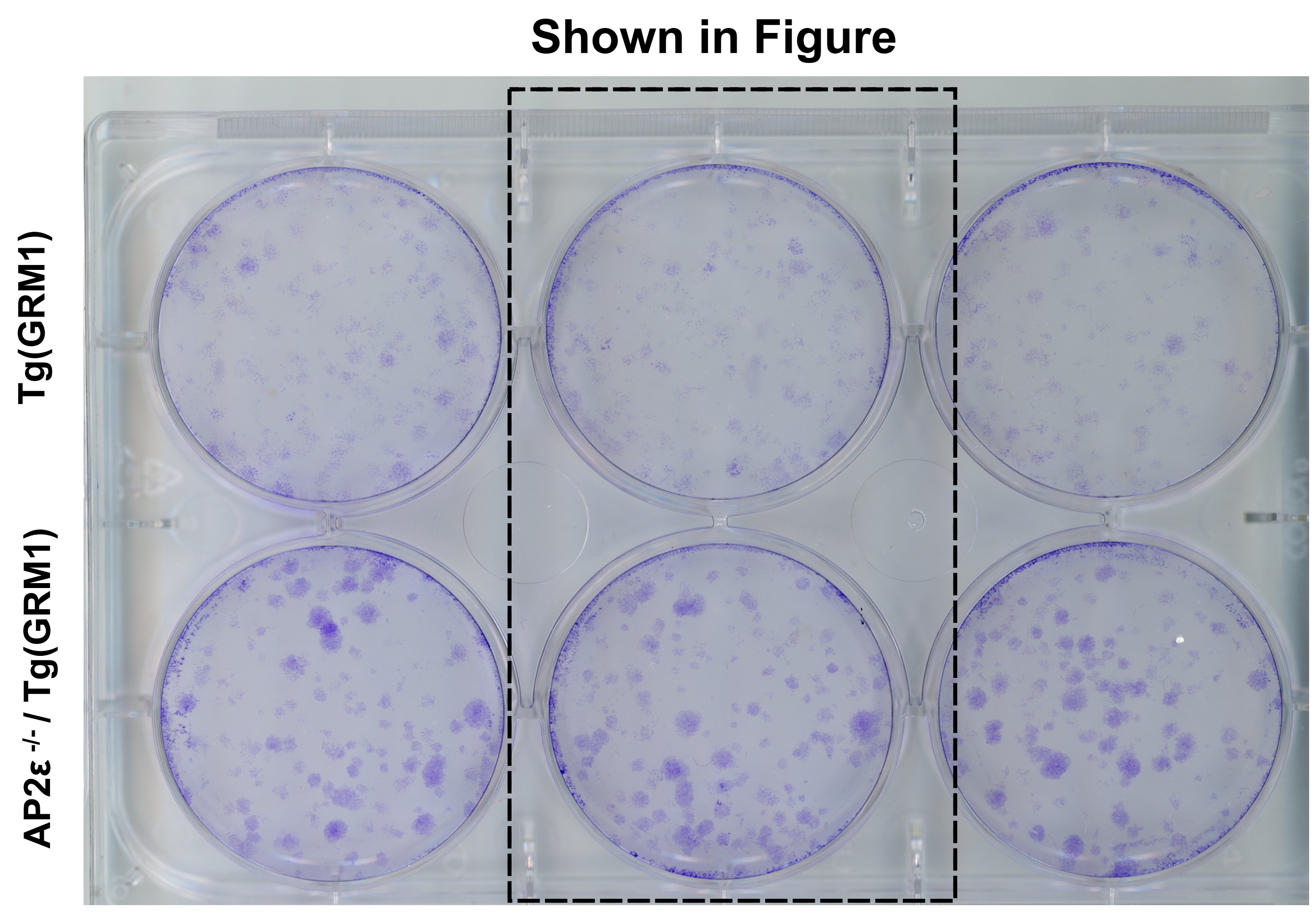

Original Data

Figure 5 A – Western Blot

MITF

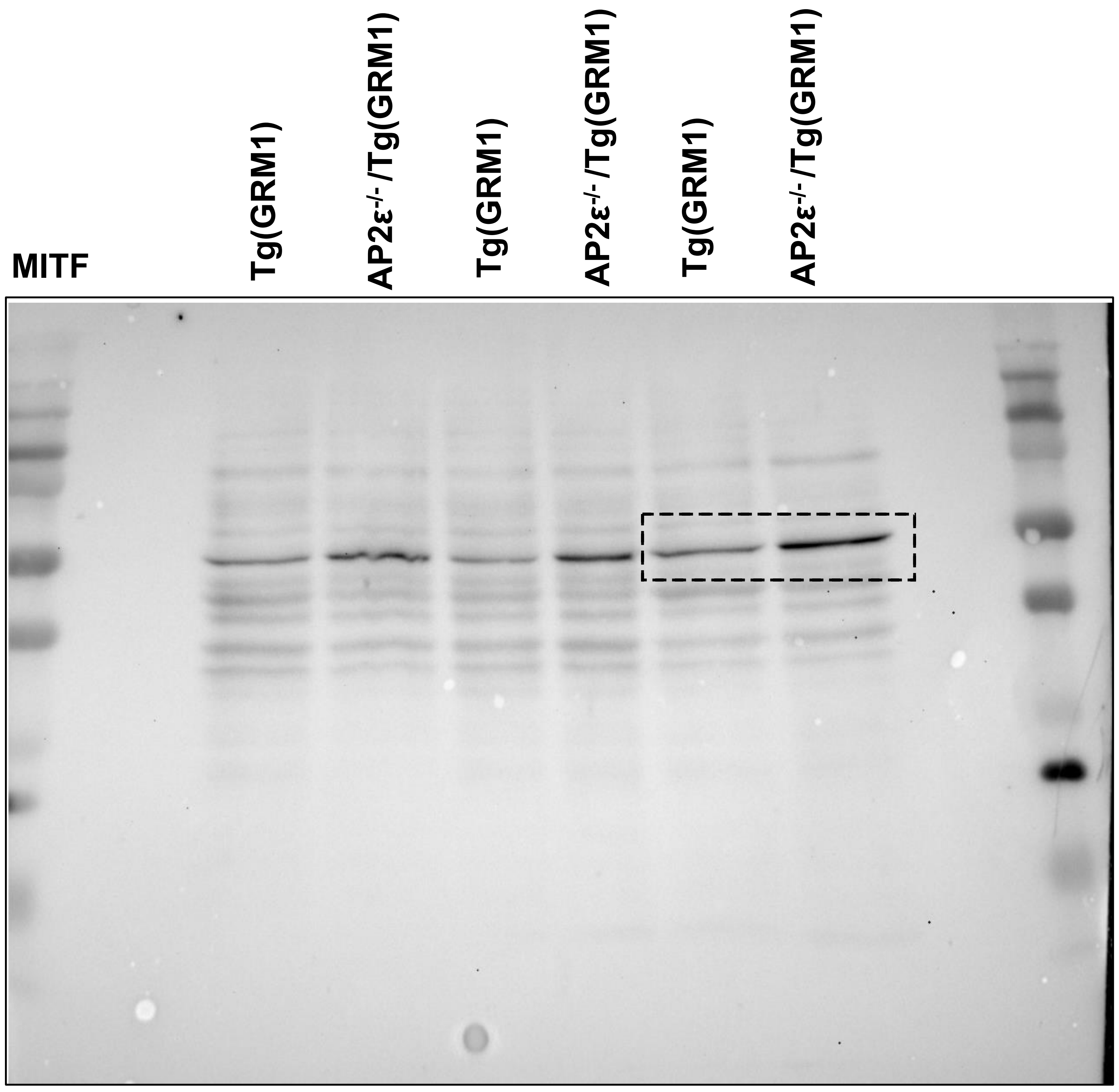

β-actin

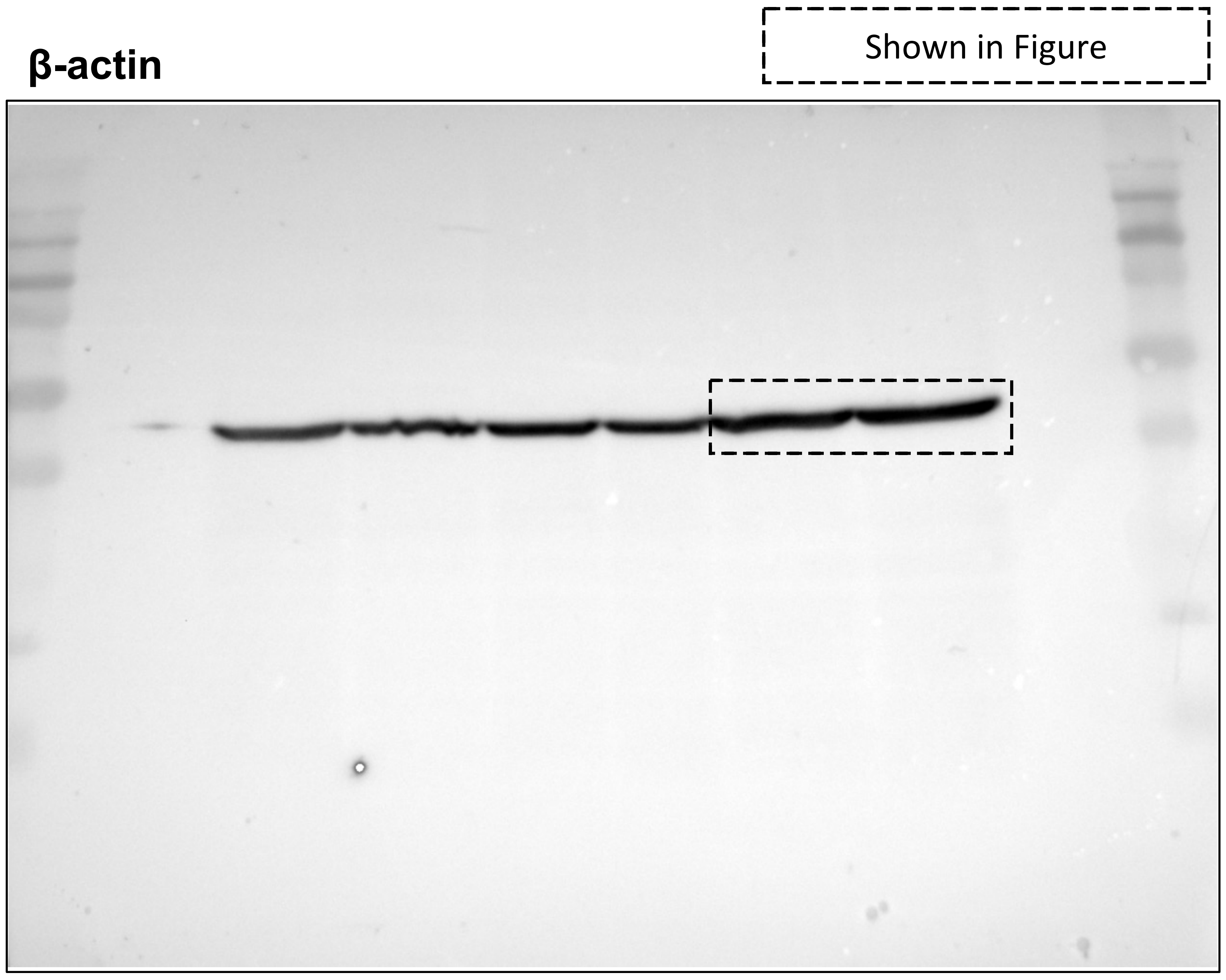

BRN2

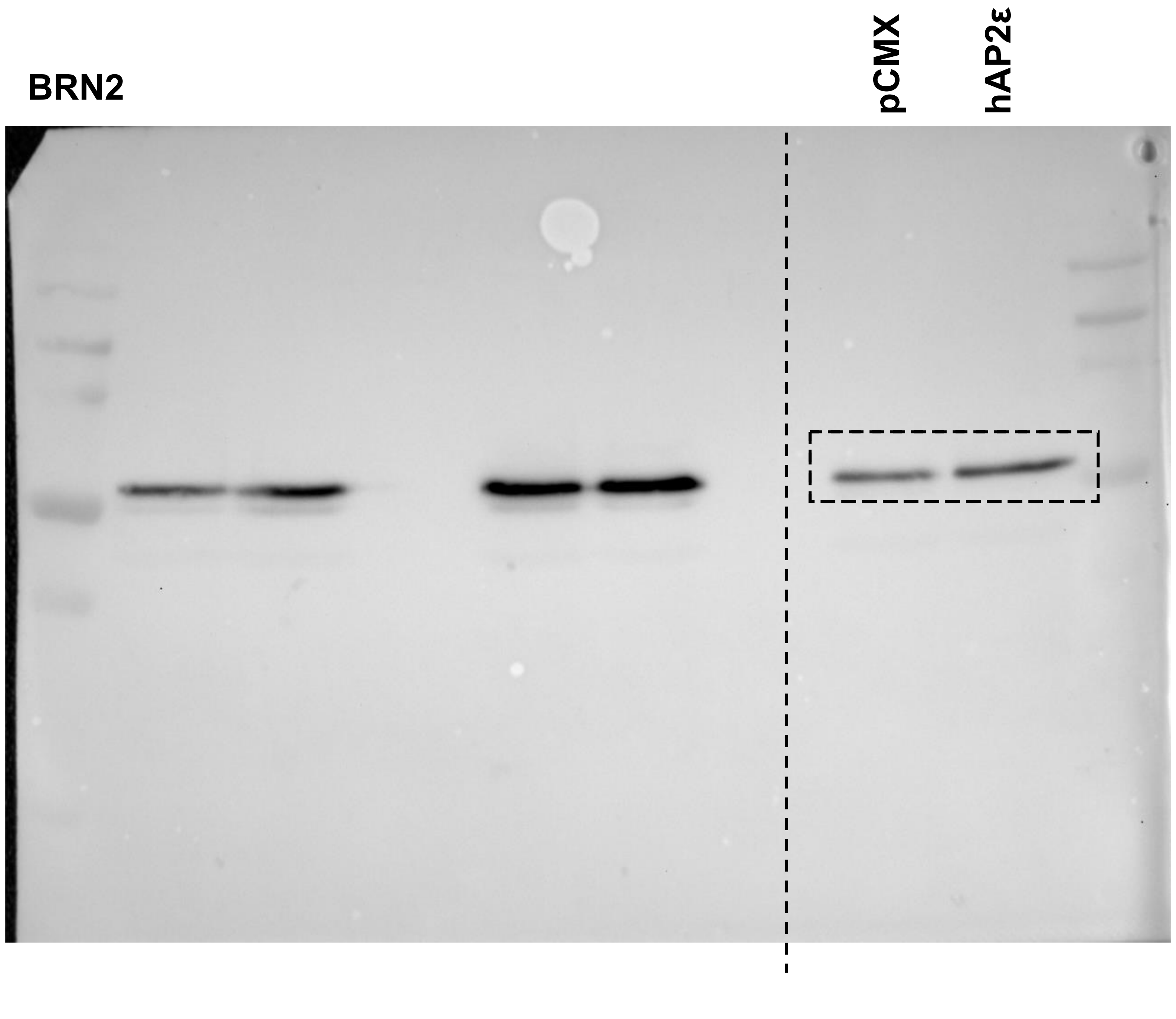

β-actin

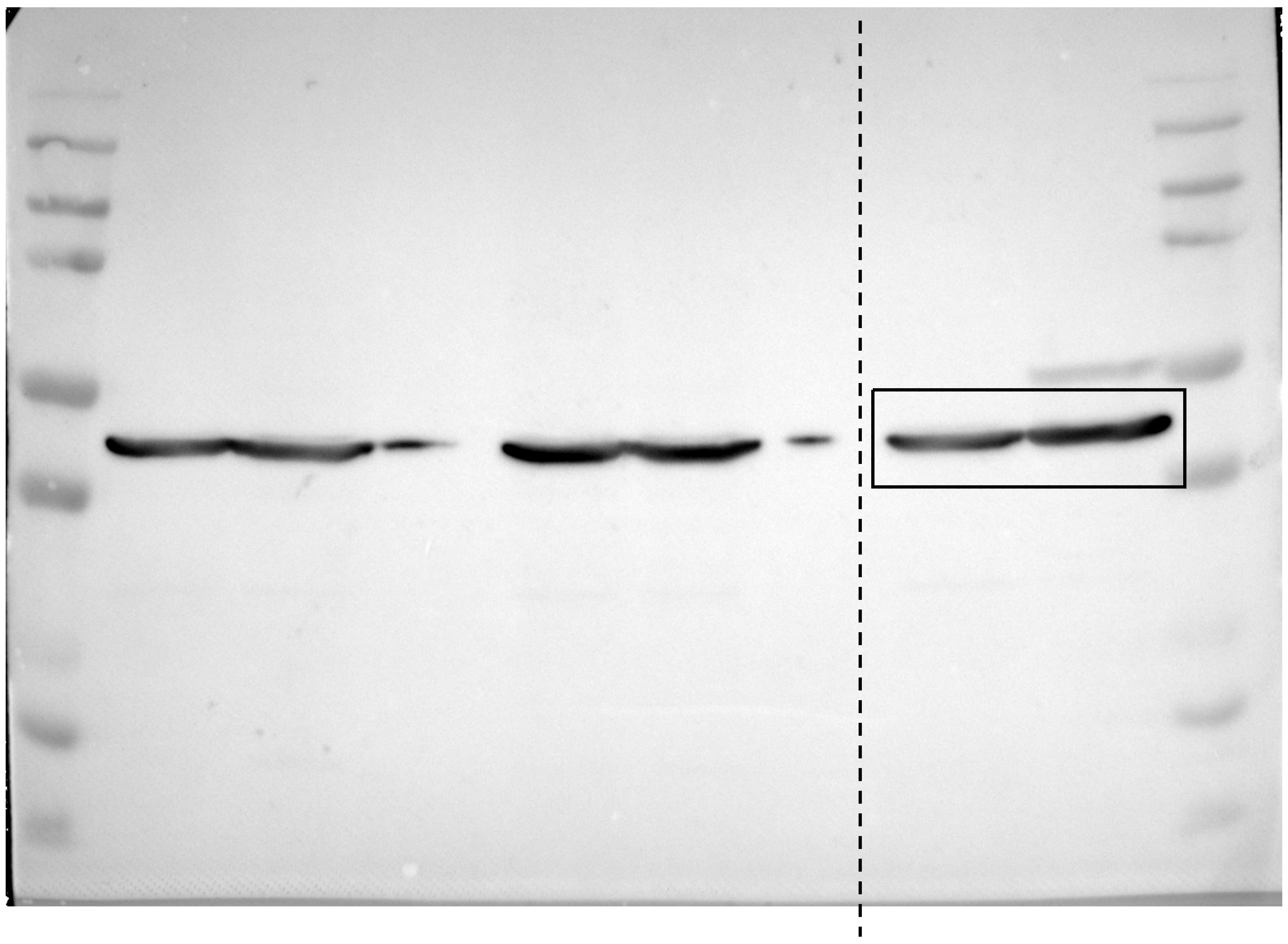

Original Data

Figure 5 A – Western Blot

MITF

pCMX hAP2ε

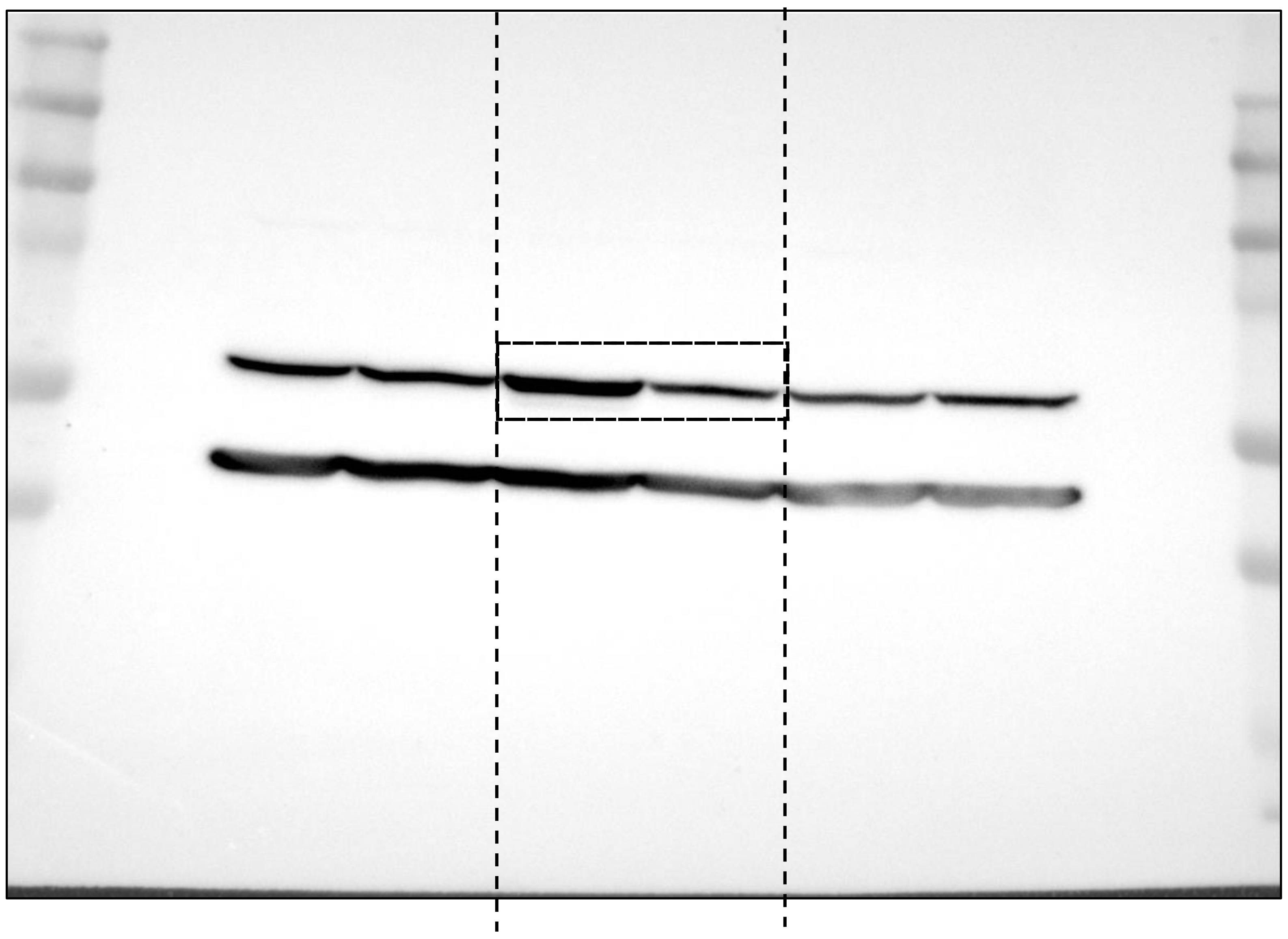

β-actin

pCMX hAP2ε

Shown in Figure

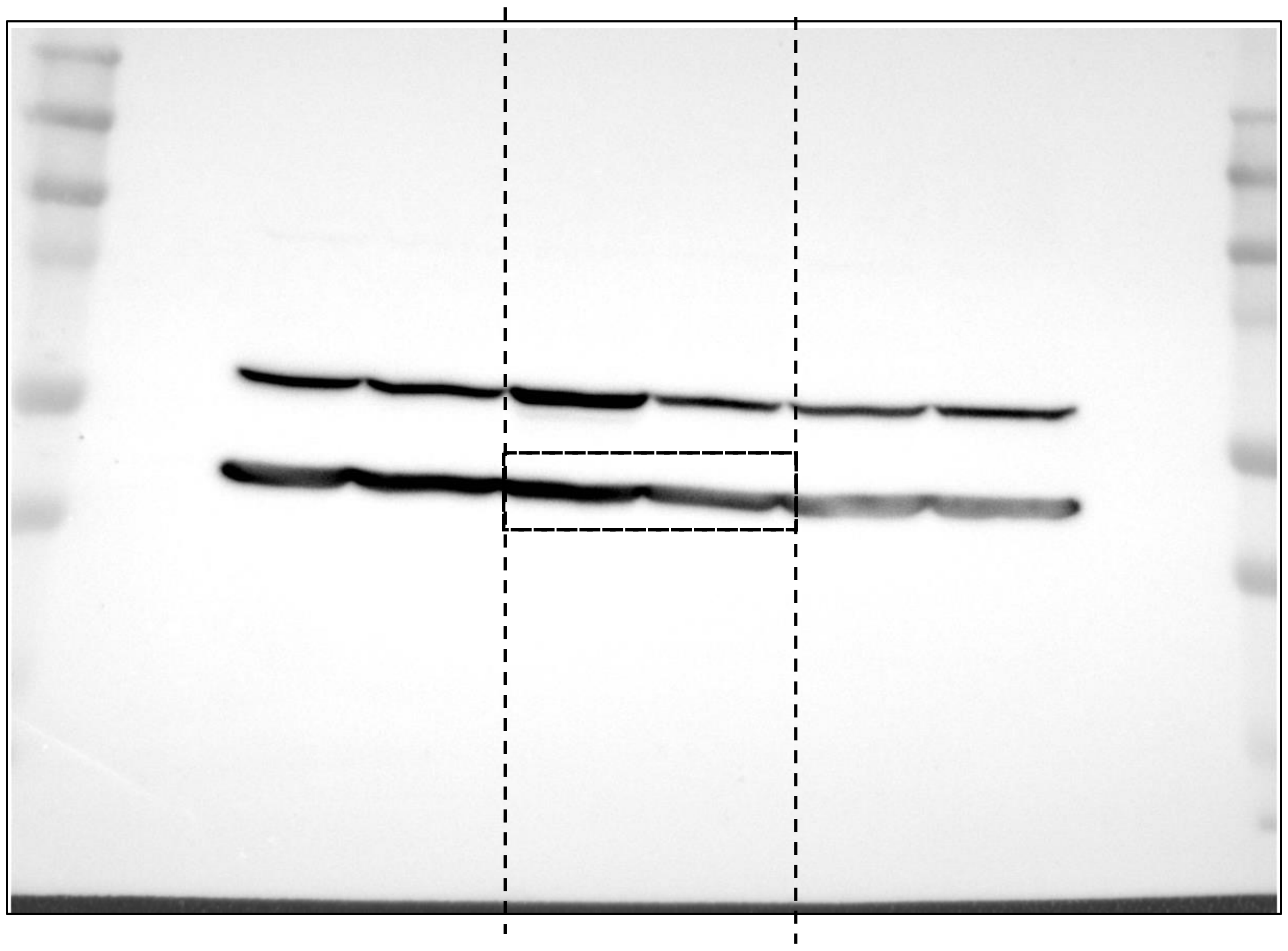

BRN2

Tg(GRM1) AP2ε$^{-/-}$/Tg(GRM1)

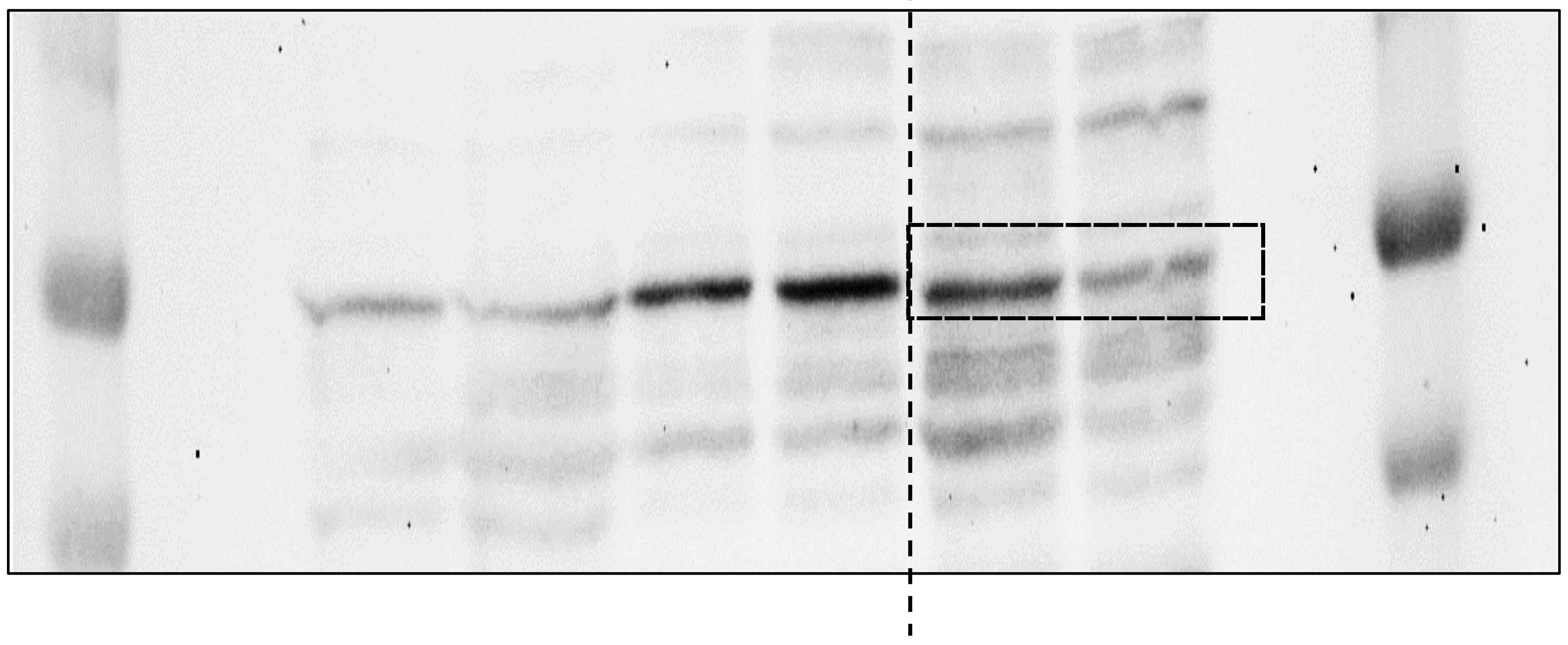

β-actin

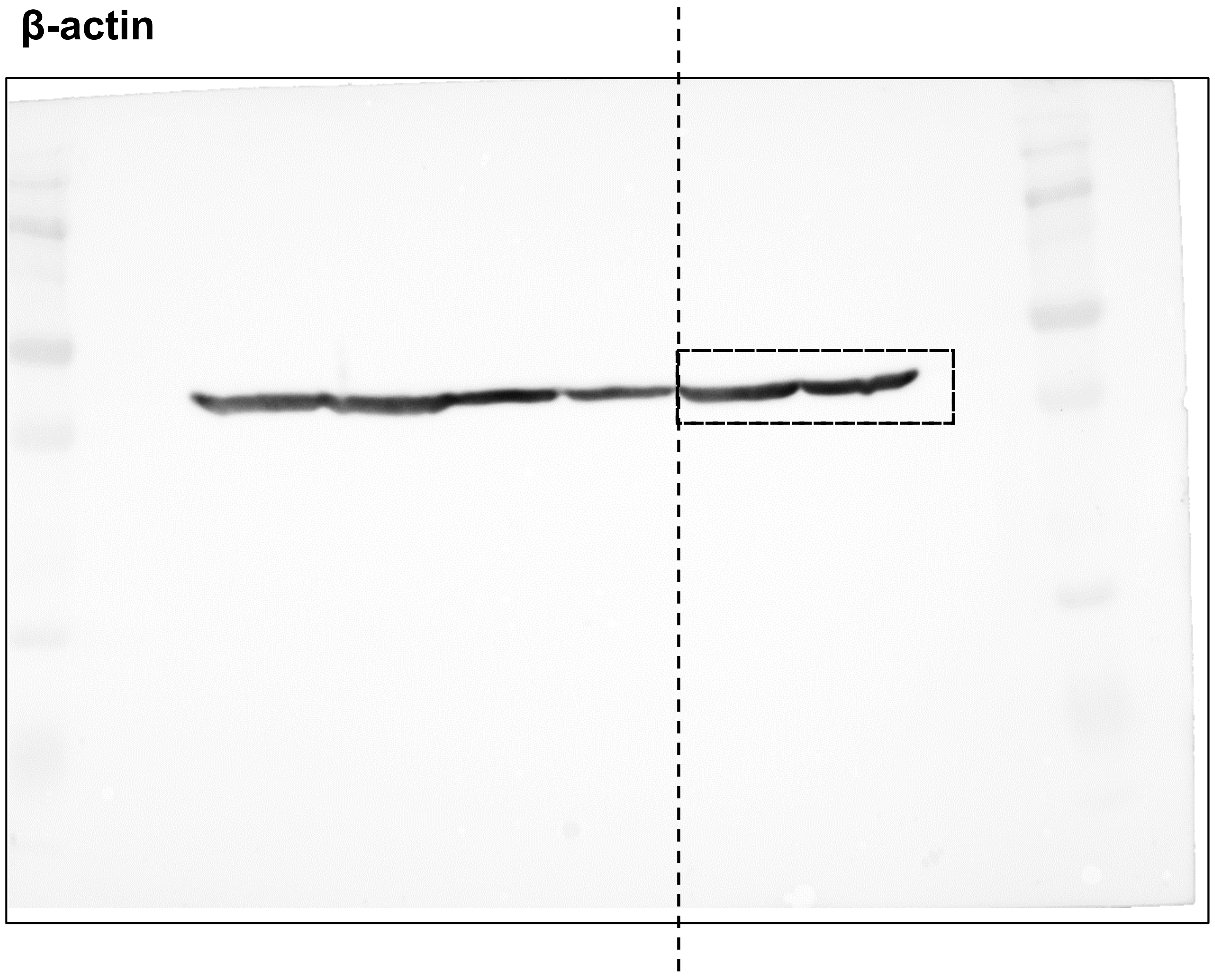

Original Data

Figure 5C/D – Western Blot

mSNAIL

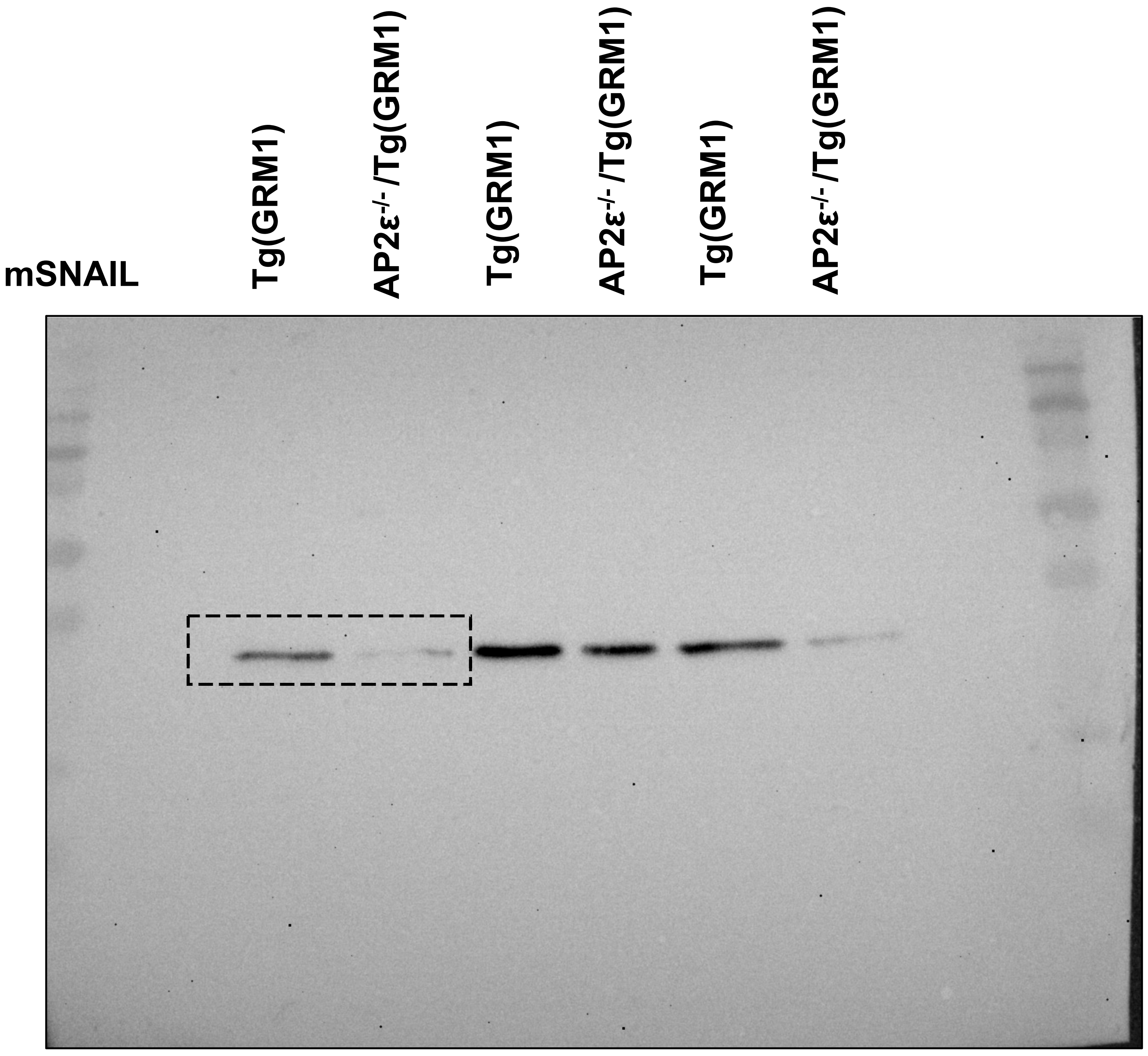

β-actin

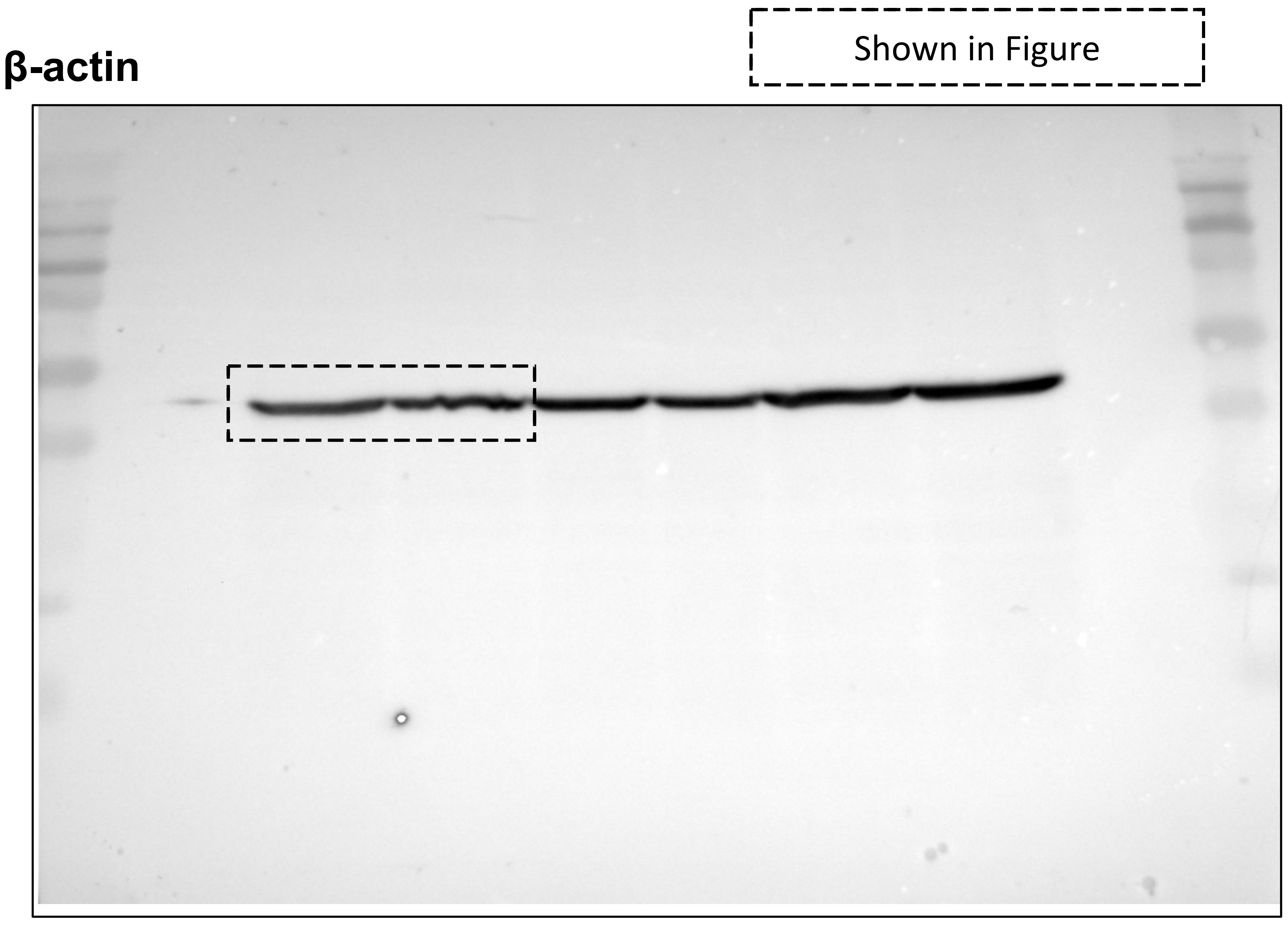

hSNAIL

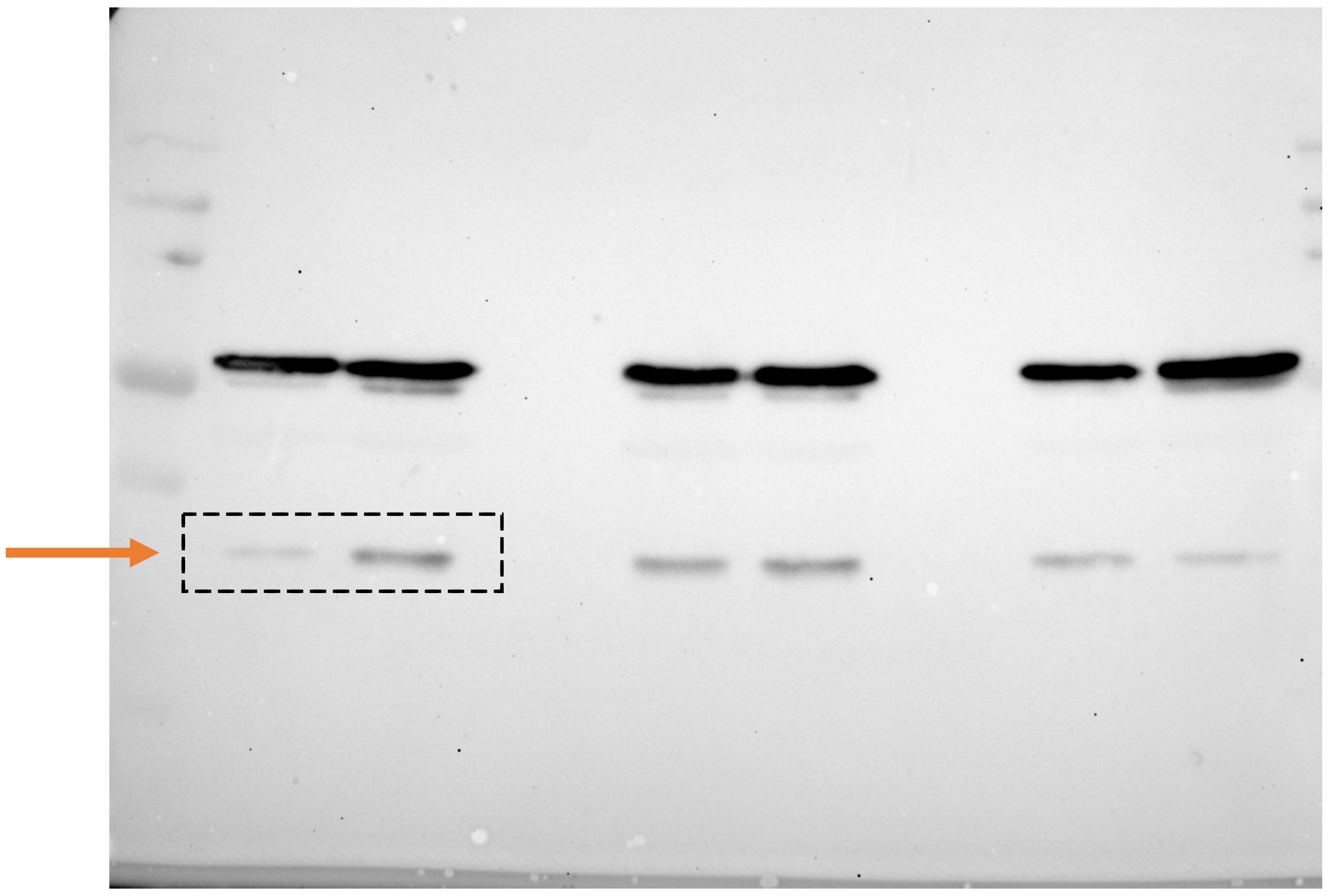

β-actin

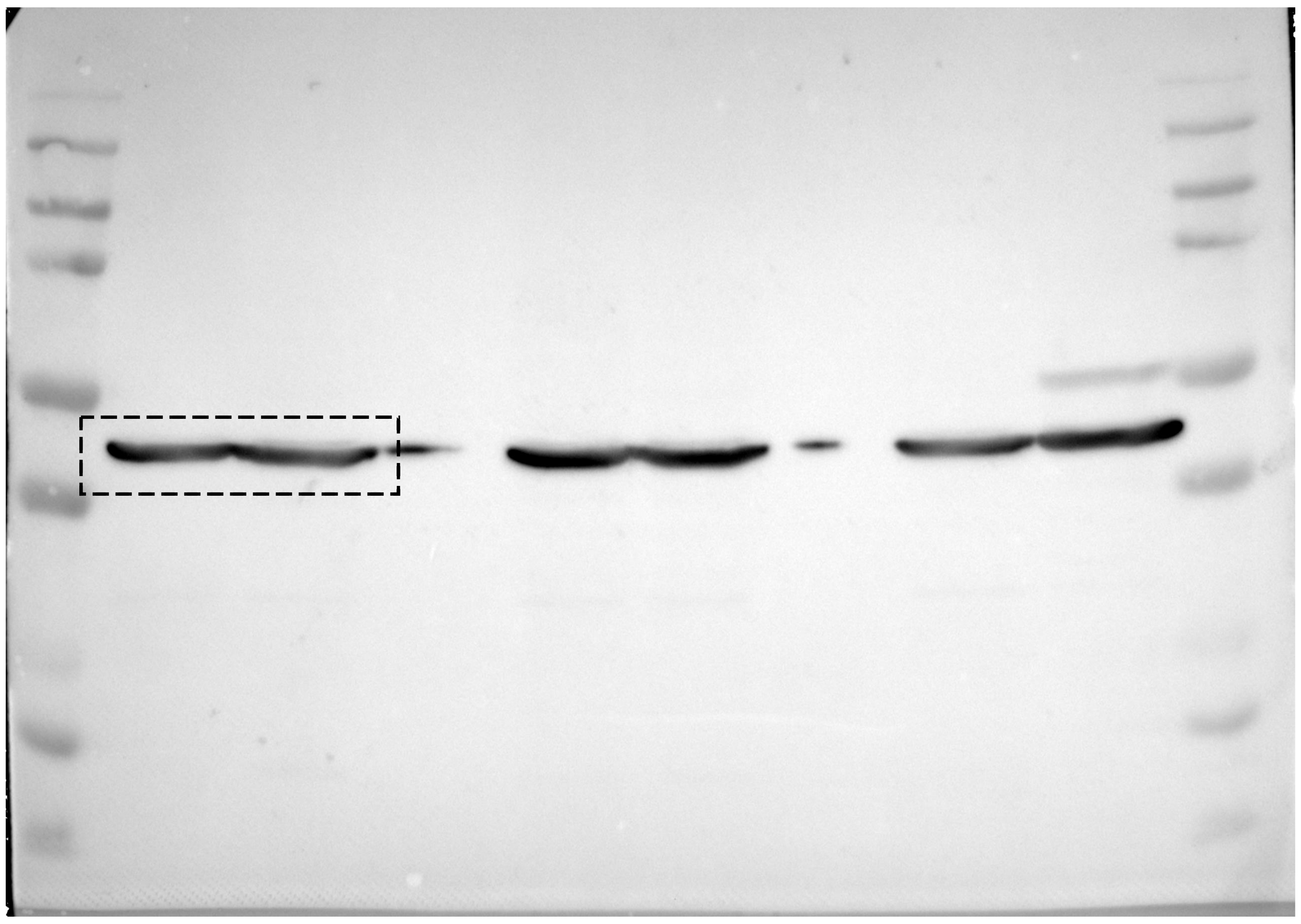

Original Data

Figure 5 E/F – Western Blot

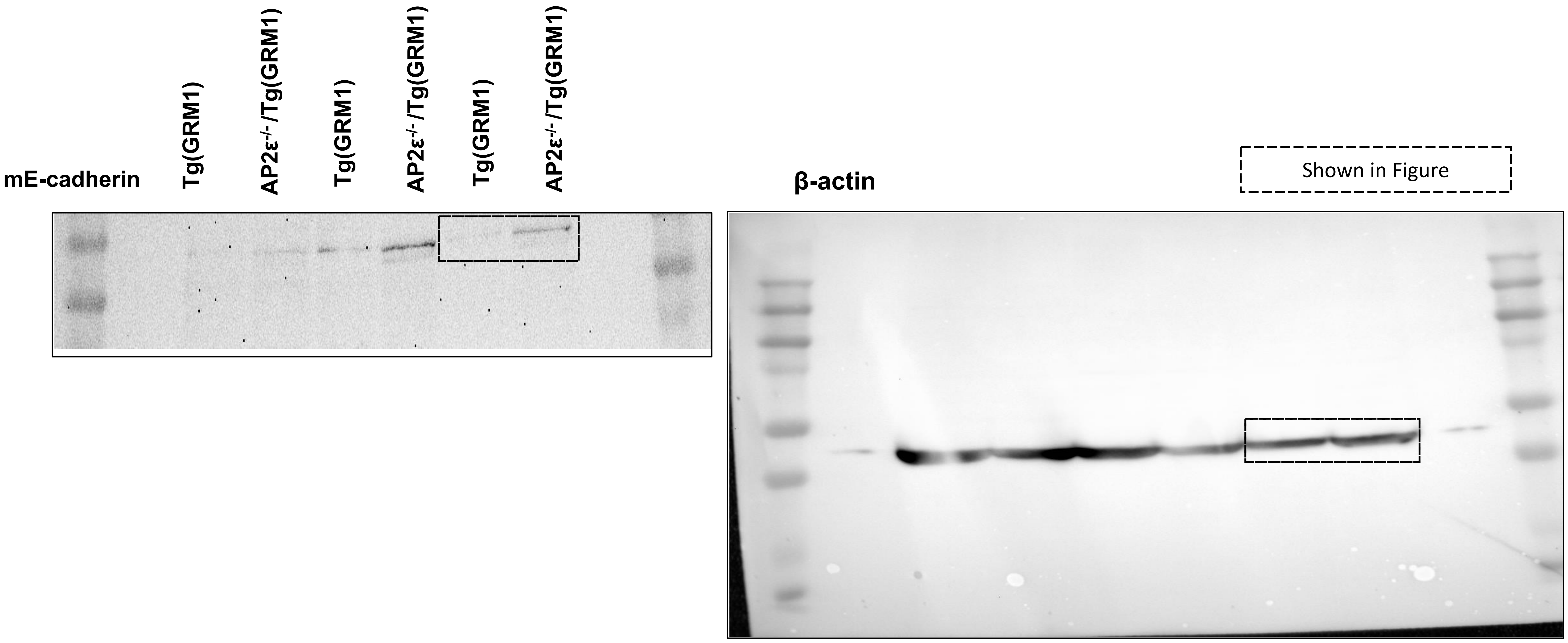

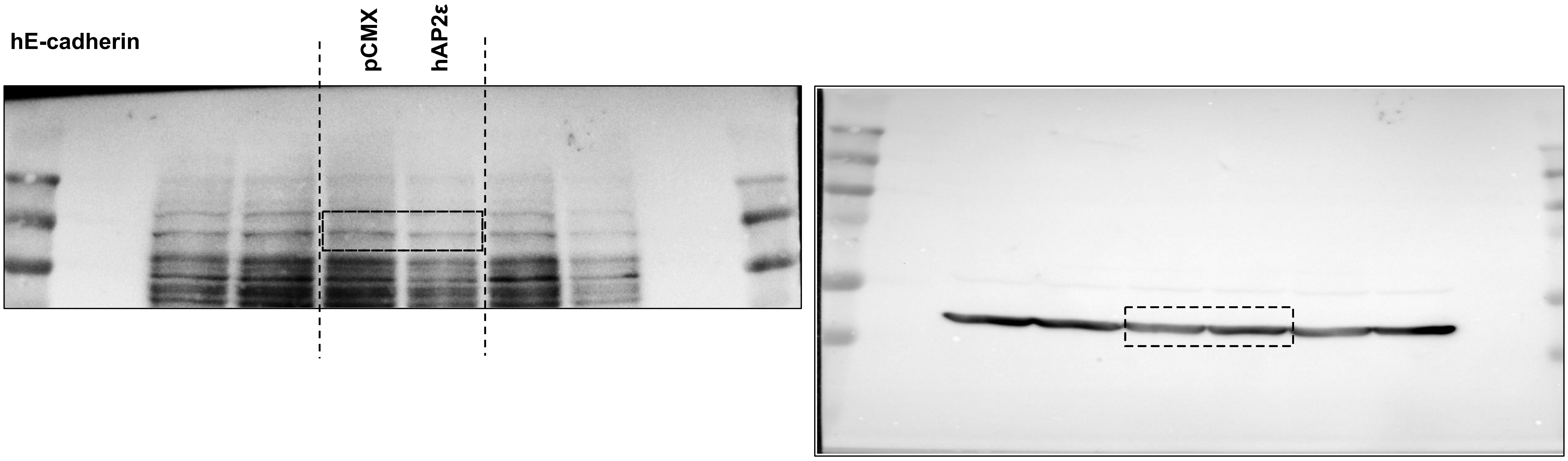

Supplement: Supplementary file 1 — Original Data [file 41419_2024_6733_MOESM1_ESM.pdf]
