# Supplementary material for: Transcription factor activating enhancer-binding protein 2ε (AP2ε) modulates phenotypic plasticity and progression of malignant melanoma
Source: Cell Death Dis. 2024 May 21;15(5):351. doi: 10.1038/s41419-024-06733-3 (PMC11109141; doi:10.1038/s41419-024-06733-3)
Supplement: Supplementary file 2 — Suplementary material and methods [file 41419_2024_6733_MOESM2_ESM.docx]

**Supplementary Material and Methods**

**FUCCI Reporter Cells**

The FUCCI (fluorescence ubiquitination-based cell cycle indicator)-labelled Mel Im cells were generated and quantified as described previously [21]. Expression of fluorescence coupled portions of the cell cycle proteins Chromatin licensing and DNA replication factor 1 (Cdt1) (red) or geminin (green) indicate either G1 or S/G2/M state of the cells. During the transition from G1 to S phase, both proteins are present and merge producing a yellow fluorescence signal.

**Real-time cell proliferation assay (RTCA)**

Real-time analysis of cell attachment was performed with the xCELLigence System (Roche, Mannheim, Germany) as described previously [23, 24]. Briefly, approximately 3 x 10^3^ primary melanoma cells deriving from AP2ε^-/-^/Tg(GRM1) and Tg(GRM1) deriving from lung metastasis, were seeded into E-Plates and monitored for 4 h. The parameter slope describes the steepness of each curve during attachment. Both cell lines were measured in triplicates in three independent experiments.

**Clonogenic assay**

Clonogenic assays were performed as described previously [25]. Briefly, 300 cells were seeded in a well of a 6-well plate, incubated for 7 days and subsequently fixed with 6 % glutaraldehyde and 0.36 % crystal violet for 30 min at room temperature. The number of colonies in each well was analyzed from scanned pictures of the plates with the “CellSens Dimension” software (Olympus K.K., Shinjuku, Tokyo, Japan). Clonogenic assays were carried out in triplicates and repeated at least three independent times.

**Boyden Chamber migration assay**

Migration assays with primary murine melanoma cells were performed using Boyden chambers containing polycarbonate filters. For this assay, primary murine melanoma cells derived from AP2ε^-/-^/Tg(GRM1) and Tg(GRM1) mice as well as the melanoma cell line Mel Im transfected with the AP2ε overexpression plasmid and the control vector pCMX, respectively, were resuspended in DMEM without fetal calf serum (FCS) and seeded in the upper compartment in a density of 4 x 10^4^ cells per chamber. The lower compartment was filled with fibroblast-conditioned medium (FKM) used as a chemoattractant. Boyden chambers were incubated for 4 h at 37 °C. Subsequently, migrated cells were fixed with methanol, stained with hematoxylin and eosin and counted with the “CellSens Dimension” software (Olympus K.K., Shinjuku, Tokyo, Japan). Experiments were carried out in triplicates and repeated at least three times.

**XTT cell viability assay**

For XTT cell viability assay, 1 x 10^3^ cells/Well were seeded in a 96-well plate. Cell viability was assessed daily over an incubation period of 7 days using the Cell Proliferation Kit II (Roche) according to the manufacturer’s instructions. A Clariostar Plus Multiplate reader (BMG Labtech, Ortenberg, Germany) was used for photometric detection. XTT Assays were carried out in triplicates and repeated at least three independent times.

**Spheroid-assay**

To generate spheroids, 100 µl of 1 % Agar / well was pipetted into the bottom of a 96-Well plate. After approximately 15 min, the cell suspension of primary AP2ε^-/-^/Tg(GRM1) and Tg(GRM1) murine melanoma cells with 4000 cells / 100 µl DMEM was pipetted on top of the agar and incubated for 72 h. After this period of time, spheroid size was measured using an Olympus IX83 microscope in combination with Olympus CellSens Dimension software (Version 2.3, Olympus, Tokyo, Japan). For migration experiments, spheroids were transferred into a 24-Well plate containing DMEM and incubated for 72 h. Migrated cells were fixed and stained with 6 % glutaraldehyde and 0.36 % crystal violet for 30 min at room temperature. To quantify distance of outgrowth, we measured the diameter of the outgrown spheroids, using the Olympus CellSens Dimension software (Version 2.3, Olympus, Tokyo, Japan).

**Cell Staining**

For better visibility of the murine cells in hydrogel culture, cells were stained with the DMSO-free “Cell Tracking Red Dye Kit” (Abcam, ab269446) according to manufacturer’s instructions.

**Hypoxia experiments**

For hypoxia experiments, cells were cultivated in a New Brunswick Galaxy 48 series incubator (Eppendorf AG, Hamburg, Germany), flushed with 0.2 % O_2_ and 8 % CO_2_. Excessive O_2_ was replaced by nitrogen. Respective controls were incubated at normoxia (21 % O_2_, 8 % CO_2_). After 24 h cells were harvested and total RNA was isolated.

**Supplementary Tables**

**Supplementary Table 1: Oligonucleotides used for real-time PCR**

| Gene | Forward Primer | Reverse Primer |
| --- | --- | --- |
| ACTB (human) | CTACGTCGCCCTGGACTTCGAGC | GATGGAGCCGCCGATCCACACGG |
| ACTB (murine) | TGGAATCCTGTGGCATCCATGAAAC | TAAAACGCAGCTCAGTAACAGTCCG |
| TFAP2a (human) | TCCGGGACTGCCCTCTCGAC | GTGACTCAGTCCCATGAAGCGC |
| TFAP2c (human) | TTGCTGCACGATCAGACAGT | ACCGGCCTCCATTTTTCGAT |
| TFAP2e (human) | GGAGTAAGGGAGGGTGGCCTCTC | GCACCAACTCCTCAGTAGCACCTC |
| TFAP2e (murine) | CACTGTGTCACACTCCCGCCG | CGCTAGGCCGCCGTAGGGGTC |
| GRM1 (murine) | GGGCAGGGAACGCCAATTCT | TGGAAGGGCTGCTGGGAGGG |
| Snail1 (human) | AGGCCCTGGCTGCTACAAG | ACATCTGAGTGGGTCTGGAG |
| Snail1 (murine) | TTCCCAAGCAGCTGGCCAGG | ACGGTTGCAGTGGGAGCAGG |
| E-Cadherin (human) | ATCCTCCGATCTTCAATCCCACCAC | GTACCACATTCGTCACTGCTACGTG |
| E-Cadherin (murine) | ACGTATCAGGGTCAAGTGCC | CCTGACCCACACCAAAGTCT |

**Supplementary Table 2: Antibodies used for immunofluorescence staining**

| **Antibody** | **Resource** | **Dilution** |
| --- | --- | --- |
| rabbit anti-AP2ε Antiserum | Anamar Medical AB, Lund, Sweden | 1:100 |
| rat anti-Ki-67 | Invitrogen eBioscience; Cat# 14-5698-82 RRID:AB_10854564 | 1:100 |
| mouse anti-HIF-1α-Alexa647 | Santa Cruz Biotechnology Cat# sc-13515; RRID:AB_627723 | 1:100 |
| Cy3-conjugated donkey anti-rat | Jackson ImmunoResearch Labs Cat# 712-165-153, RRID:AB_2340667 | 1:400 |
| Alexa488-conjugated goat anti-mouse | Thermo Fisher Scientific Cat# A-11008, RRID:AB_143165 | 1:400 |
| Alexa555-conjugated goat anti-rabbit | Thermo Fisher Scientific Cat# A32732, RRID:AB_2633281 | 1:400 |
