# Supplementary material for: Transcription factor activating enhancer-binding protein 2ε (AP2ε) modulates phenotypic plasticity and progression of malignant melanoma
Source: Cell Death Dis. 2024 May 21;15(5):351. doi: 10.1038/s41419-024-06733-3 (PMC11109141; doi:10.1038/s41419-024-06733-3)
Supplement: Supplementary file 3 — Supplementary Figures [file 41419_2024_6733_MOESM3_ESM.pdf]

Supplementary Figure S1

Validation of primary murine melanoma cells derived from Tg(GRM1) and AP2ε$^{-/-}$/Tg(GRM1) mice.

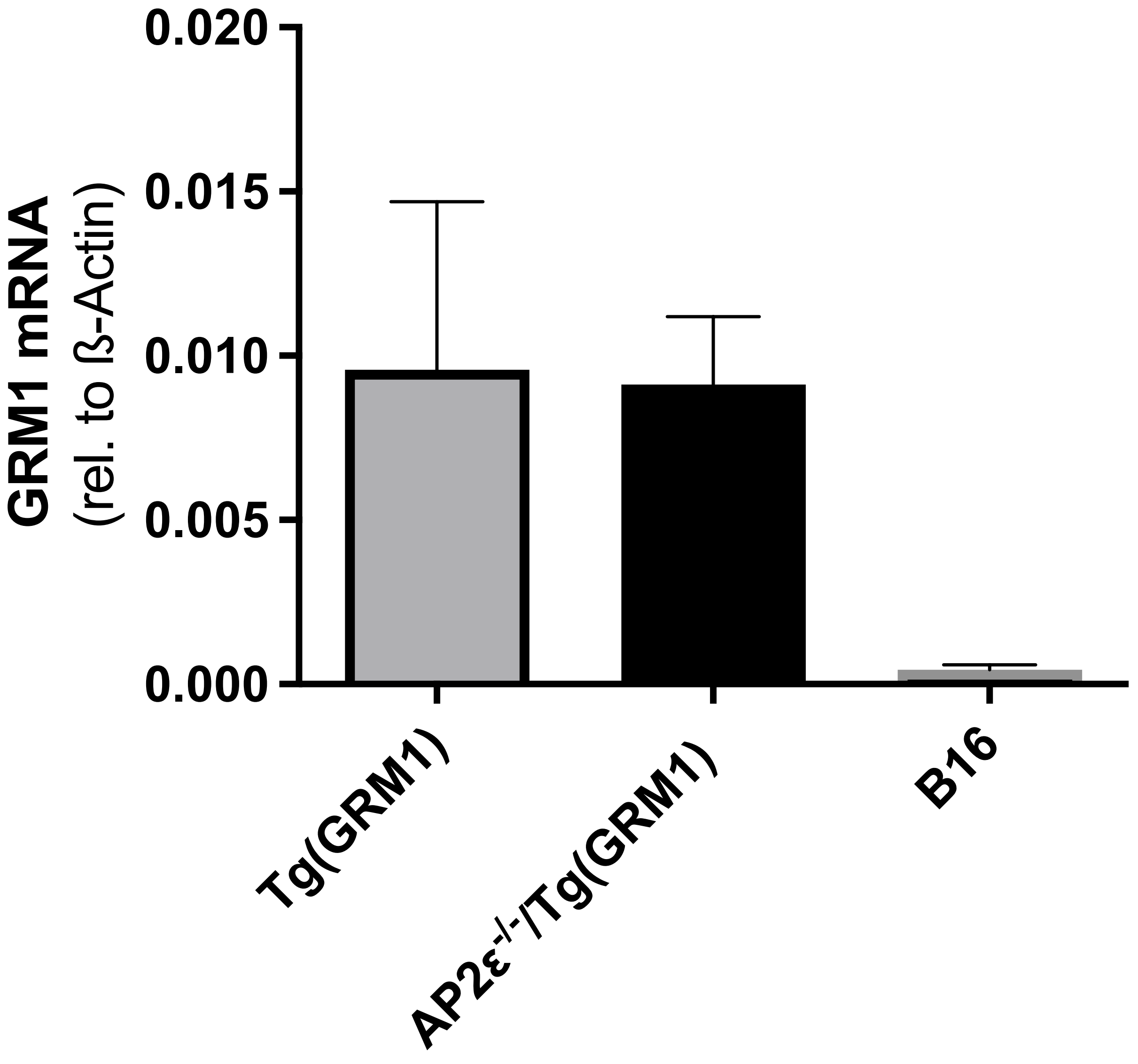

Validation of primary murine melanoma cells derived from Tg(GRM1) and AP2ε-/-/Tg(GRM1) mice: mRNA-expression analysis for GRM1 in Tg(GRM1) (*n=3*) and AP2ε$^{-/-}$/Tg(GRM1) (*n=3*) cells; B16 (*n*=5) as a control. Data information: Data are represented as mean ± SEM.

Supplementary Figure S2

Quantification of AP2ε-positive cells

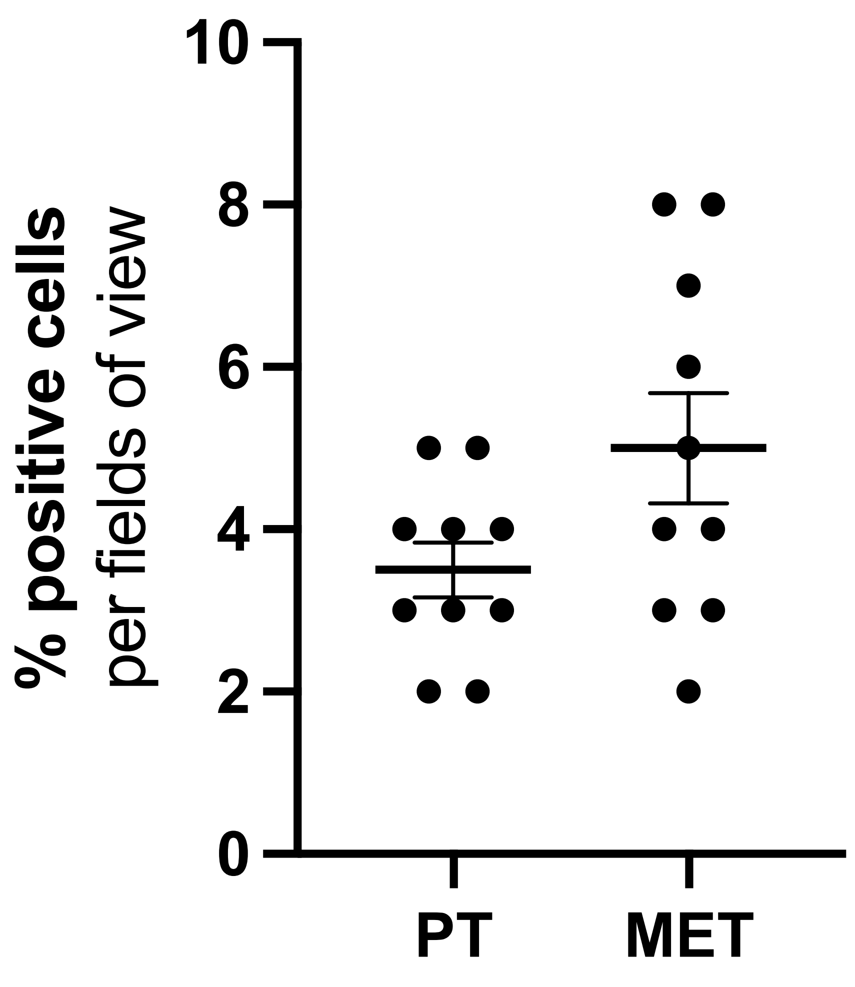

Quantification of AP2ε-positive cells in Primary tumors (PT) and Metastasis (MET)

Quantification of percentage of cells with AP2ε expression. Data are represented as mean ± SEM.

Supplementary Figure S3

Bioinformatical analysis of quiescent comparing cycling cells (GSE174520)

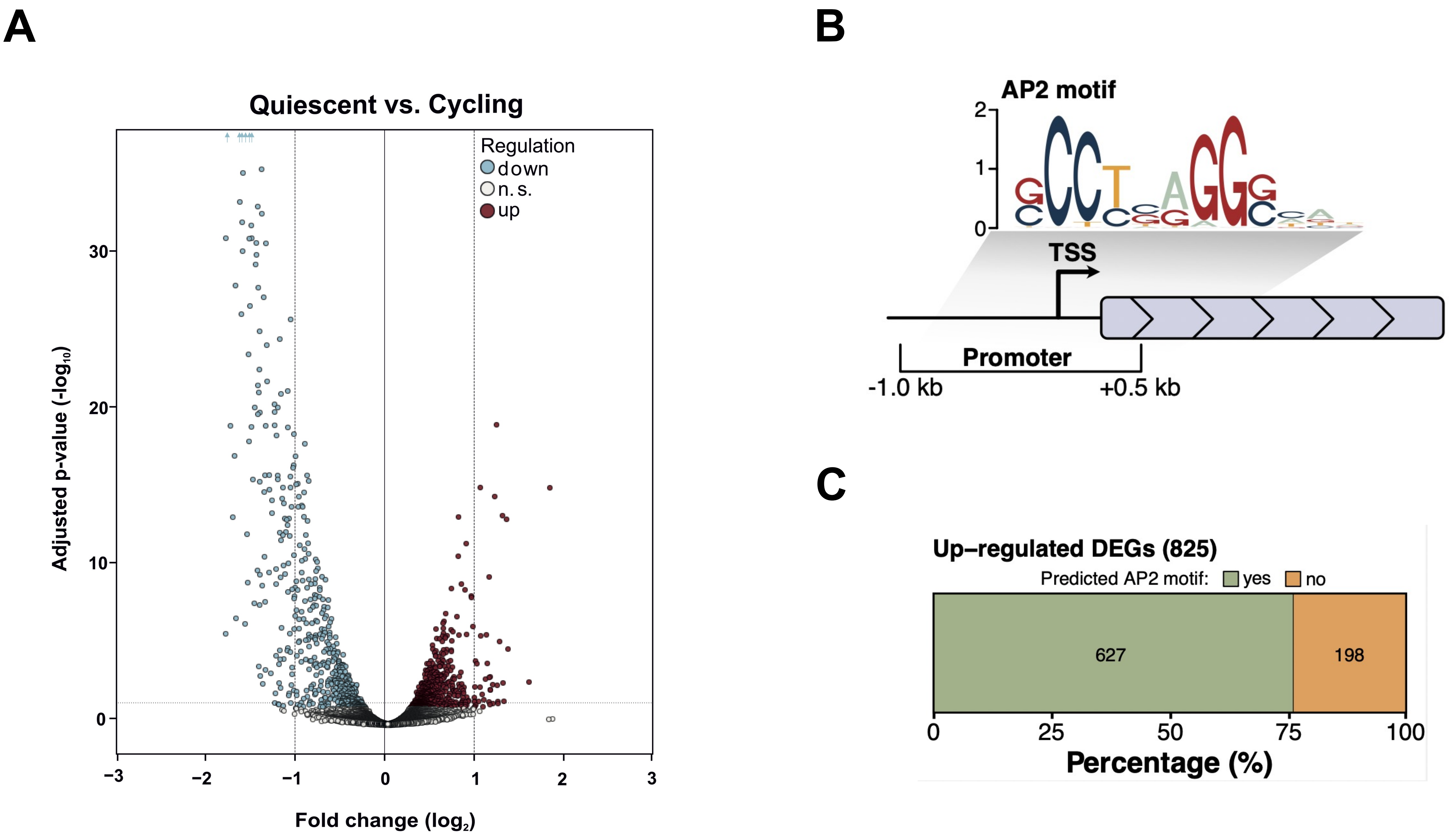

Figure S2. Bioinformatical analysis of quiescent comparing cycling cells (GSE174520)

A Volcano Plot depicting significant deregulated genes (DEGs) in quiescent cells compared to cycling cells. **B** Scheme depicting AP2-transcription factor binding motif +0,5kb to -1 kb upstream of the transcription start (TSS) **C** Quantification of up-regulated DEGs with or without a predicted AP2 motif.

Supplementary Figure S4

AP2α expression is elevated in AP2ε-/-/Tg(GRM1) Tissue

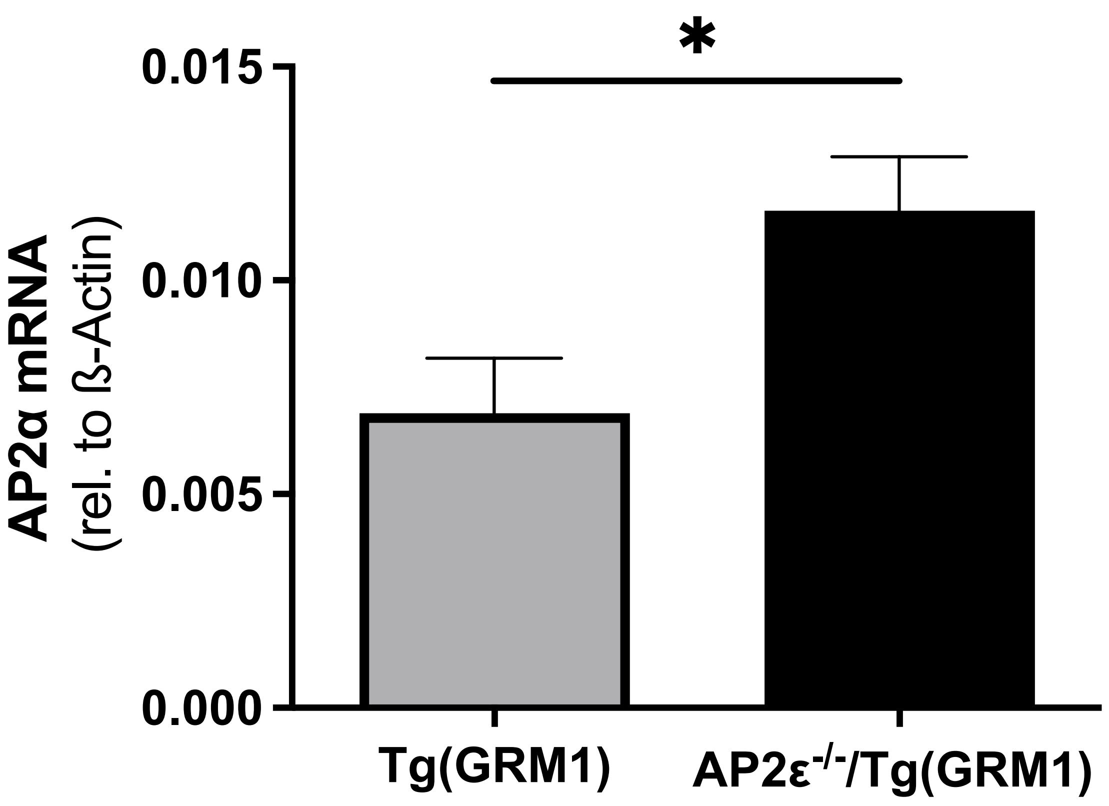

AP2α expression is elevated in AP2ε-/-/Tg(GRM1) Tissue: mRNA-expression analysis for AP2α in AP2ε$^{-/-}$/Tg(GRM1) tissue; Tg(GRM1) (*n=7*) and AP2ε$^{-/-}$/Tg(GRM1) (*n=8*) Data information: Data are represented as mean ± SEM; *: $p < 0.05$ (Two-tailed Student's t-test)
